# Enhancing Mental and Physical Health of Women through Engagement and Retention (EMPOWER): a protocol for a program of research

**DOI:** 10.1186/s13012-017-0658-9

**Published:** 2017-11-07

**Authors:** Alison B. Hamilton, Melissa M. Farmer, Tannaz Moin, Erin P. Finley, Ariel J. Lang, Sabine M. Oishi, Alexis K. Huynh, Jessica Zuchowski, Sally G. Haskell, Bevanne Bean-Mayberry

**Affiliations:** 1 Veterans Affairs Greater Los Angeles Healthcare System, Los Angeles, CA USA; 2 Veterans Affairs Health Services Research and Development Center for the Study of Healthcare Innovation, Implementation & Policy, Los Angeles, California USA; 3 David Geffen School of Medicine, University of California Los Angeles, Los Angeles, CA USA; 4 South Texas Veterans Health Care, San Antonio, TX USA; 5 University of Texas Health Science Center, San Antonio, TX USA; 6 VA San Diego Healthcare System, San Diego, CA USA; 7 University of California San Diego, San Diego, CA USA; 8 VA Connecticut Healthcare System, West Haven, CT USA; 9 Yale School of Medicine, Yale University, New Haven, CT USA

**Keywords:** Women veterans, Patient engagement, Replicating effective programs, Implementation science, Stepped wedge, Diabetes prevention, Cardiovascular risk reduction, Collaborative care

## Abstract

**Background:**

The Enhancing Mental and Physical health of Women through Engagement and Retention or EMPOWER program represents a partnership with the US Department of Veterans Health Administration (VA) Health Service Research and Development investigators and the VA Office of Women’s Health, National Center for Disease Prevention and Health Promotion, Primary Care-Mental Health Integration Program Office, Women’s Mental Health Services, and the Office of Patient Centered Care and Cultural Transformation. EMPOWER includes three projects designed to improve women Veterans’ engagement and retention in evidence-based care for high-priority health conditions, i.e., prediabetes, cardiovascular, and mental health.

**Methods/Design:**

The three proposed projects will be conducted in VA primary care clinics that serve women Veterans including general primary care and women’s health clinics. The first project is a 1-year quality improvement project targeting diabetes prevention. Two multi-site research implementation studies will focus on cardiovascular risk prevention and collaborative care to address women Veterans’ mental health treatment needs respectively. All projects will use the evidence-based Replicating Effective Programs (REP) implementation strategy, enhanced with multi-stakeholder engagement and complexity theory. Mixed methods implementation evaluations will focus on investigating primary implementation outcomes of adoption, acceptability, feasibility, and reach. Program-wide organizational-, provider-, and patient-level measures and tools will be utilized to enhance synergy, productivity, and impact. Both implementation research studies will use a non-randomized stepped wedge design.

**Discussion:**

EMPOWER represents a coherent program of women’s health implementation research and quality improvement that utilizes cross-project implementation strategies and evaluation methodology. The EMPOWER Quality Enhancement Research Initiative (QUERI) will constitute a major milestone for realizing women Veterans’ engagement and empowerment in the VA system. EMPOWER QUERI will be conducted in close partnership with key VA operations partners, such as the VA Office of Women’s Health, to disseminate and spread the programs nationally.

**Trial registration:**

The two implementation research studies described in this protocol have been registered as required:

Facilitating Cardiovascular Risk Screening and Risk Reduction in Women Veterans: Trial registration NCT02991534, registered 9 December 2016.

Implementation of Tailored Collaborative Care for Women Veterans: Trial registration NCT02950961, registered 21 October 2016.

**Electronic supplementary material:**

The online version of this article (10.1186/s13012-017-0658-9) contains supplementary material, which is available to authorized users.

## Background

Women Veterans are the fastest growing segment of Veterans Health Administration (VA) users, their population increasing by 80% from 2003 to 2012 [1]. This dramatic growth has created major challenges for women Veterans seeking care in the VA healthcare system, as a numerical minority with distinctive physical and mental health care needs, and for VA, whose providers have varying and often limited exposure to female patients. Ample research suggests that a sizeable proportion of women Veterans have yet to feel that VA is their “medical home,” although the introduction of designated women’s health (WH) providers has resulted in slightly improved patient experiences of care [2]. Despite many improvements [3], gender disparities persist in diabetes and cardiovascular (CV) risk factor control [4, 5], and rates of depression, anxiety, and mental health comorbidity are disproportionately high among women Veterans [6]. Women Veterans’ high rate of attrition from VA care [7, 8], combined with persistent organizational barriers to care (e.g., lack of gender-specific services, care fragmentation) [9–12], indicate that VA needs to identify innovative ways to improve quality and patient centeredness of care for women, more effectively engage women in that care, and sustain their engagement to improve their satisfaction and optimize health outcomes.

Enhancing Veteran engagement with health care has been identified as a key strategic goal for VA in advancing “health care that is personalized, proactive, and patient-driven, and engages and inspires Veterans to their highest possible level of health and well-being.” VA’s commitment to patient-centered approaches builds on findings that individuals who actively participate in their health care are more satisfied, and have better outcomes at reduced costs: the “triple aim” of healthcare systems [13]. Promoting patient engagement in care may be especially useful in improving quality of care among high risk or under-represented populations, such as women Veterans [14].

Implementation research is designed to address gaps in care, and to speed the process by which evidence-based treatment models are adopted in routine care. The Enhancing Mental and Physical health of Women through Engagement and Retention (EMPOWER) Quality Enhancement Research Initiative (QUERI) was designed to implement innovative care models in VA women’s health, in order to improve women Veterans’ engagement and retention in evidence-based care. In developing EMPOWER, we first consulted with policy partners in Women’s Health Services’ (WHS) and Mental Health Services (MHS) to ascertain their priorities for VA women’s health (WH) implementation research. They encouraged us to identify models that address multifactorial health conditions (as opposed to single disease models) and risk factors among women Veteran VA users, as well as approaches that offer strategic planning, support, and flexibility to providers. We examined women Veterans’ top health conditions, reviewed the evidence for treating those conditions, and identified appropriate projects and leads. We arrived at a combination of projects that target clinical areas where gender disparities persist in VA (CV and prediabetes) and where the conditions are more prevalent among women Veterans (depression and anxiety). We then expanded discussions to include partners in areas specific to our projects and impact goal: National Center for Health Promotion and Disease Prevention (NCP), Primary Care-Mental Health Integration (PC-MHI) Program Office, and the Office of Patient Centered Care and Cultural Transformation (OPCC&CT). Leaders from all of these offices serve on our Strategic Advisory Group. Moreover, our QUERI has been strengthened from its inception by invaluable input of women Veterans themselves. Specifically, a newly formed Women Veteran Patient Advisory Council agreed to serve as an implementation partner for EMPOWER; Dr. Hamilton will serve as civilian member of the Council and will use principles of patient engagement in research [15] to guide collaboration and involvement of the Council. This is one of the first examples of a formalized research partnership with women Veterans to enhance quality improvement and research objectives in VA. Our strong partnerships with VA policy and operations stakeholders will directly contribute to policy impacts and also accelerate existing efforts to better engage Veterans in VA health services research.

As described in the Implementation Core below, we have taken an integrated, cross-Program approach to conducting our projects, using one consistent evidence-based implementation strategy, highlighting multilevel stakeholder engagement and applying complexity theory. Our goal is to improve women Veterans’ engagement and retention through implementation of evidence-based care models for three high-priority health conditions, i.e., prediabetes, cardiovascular disease, and mental health. To achieve this goal, we propose a cohesive portfolio of projects with the following aims:

1. To use an evidence-based implementation strategy that emphasizes local tailoring of care models, multilevel stakeholder engagement, and systematic evaluation of complex implementation processes in order to enrich organizational capacity for innovations in women Veterans’ VA health care
2. To implement personalized, proactive, patient-centered innovations in VA women’s health that are acceptable, feasible, satisfactory, relevant, and effective for both providers and patients, thereby encouraging women Veterans’ engagement and retention *and* sustainability of the innovations
3. To generate implementation “playbooks” for our partners that are scalable and serve as guidance for future implementation of a broader array of evidence-based women’s health programs and policy.

## Methods/design

### Overview of the three projects

EMPOWER QUERI aims will be realized through the conduct of three projects sharing a core conceptual framework and methodological approach. The first project entitled, “Tailoring VA’s Diabetes Prevention Program to Women Veterans’ Needs” is a 1-year QI project to be conducted in VA Greater Los Angeles women’s health clinics. Women Veterans with prediabetes will be offered the choice of either an in-person, peer-led, or online gender-specific, evidence-based diabetes prevention program (DPP) to address their risk behaviors and prediabetes (Dr. Tannaz Moin, PI; Dr. Sally Haskell, Co-PI). The second project entitled “Facilitating Cardiovascular Risk Screening and Risk Reduction in Women Veterans” (known as CV Toolkit) will increase identification of CV risk among women Veterans, enhance patient/provider communication and shared decision-making about CV risk, and provide a supportive, coordinated health coaching intervention to facilitate women Veterans’ engagement and retention in appropriate health services (Dr. Bevanne Bean-Mayberry, PI; Dr. Melissa Farmer, Co-PI). The third project, entitled, “Implementation of Tailored Collaborative Care for Women Veterans” (CCWV) will evaluate implementation of an evidence-based collaborative care model tailored to enhance provider- and system-level capabilities to address women Veterans’ anxiety and depression treatment needs, thereby improving organizational primary care-mental health integration (PC-MHI) effectiveness and women Veterans’ engagement and retention in PC-MHI (Dr. Alison Hamilton, PI; Dr. Ariel Lang, Co-PI). Details about each project are included in the online supplements. We anticipate that this use of a core framework and shared design elements will contribute to implementation science by allowing for direct comparison of the interdependencies between innovations, stakeholder and patient perspectives and needs, and organizational capacity for change in complex implementation efforts.

### Implementation core

#### Conceptual framework

**Fig. 1:**
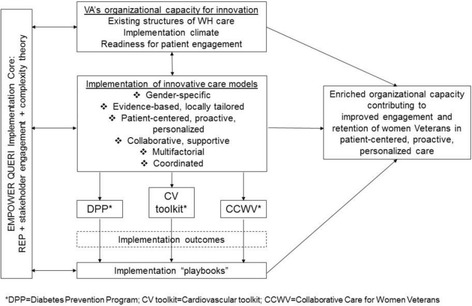
EMPOWER QUERI Conceptual Framework

This QUERI Program focuses on strengthening WH organizational capacity for innovation in patient-centered care, using an implementation core and three projects as a collective platform for examining how particular characteristics of care models contribute to providers’ ability to utilize the models and to patients’ engagement and retention in the models. As depicted in our conceptual framework (Fig. 1), the implementation core forms the backbone of our program, relies on an evidence-based implementation strategy, Replicating Effective Programs (REP), and emphasizes multilevel stakeholder engagement and complexity theory. The core will examine organizational capacity for innovation in the context of local WH care arrangements, which we know to be quite diverse, as well as implementation constructs such as climate and leadership, and novel constructs such as organizational readiness for patient engagement and implementation citizenship. Knowledge of organizational capacity will inform implementation of innovative care models. Implementation outcomes across projects, actively supported by the implementation core, will contribute to development of Implementation Playbooks—brief, user-friendly summations of implementation targets, processes, outcomes, and recommendations for scale-up and spread [16]. Together, these activities will contribute to our ultimate goal of enriching VA’s organizational capacity to engage and retain women Veterans in patient-centered, proactive, personalized evidence-based care.

#### Implementation strategy: Replicating Effective Programs (REP)

We will use REP [17] across all projects to maximize the implementation science knowledge that will be generated by a common approach. Grounded in theories of Diffusion of Innovation and Social Learning, REP was selected because of its strong evidence base and application in VA health services research [18]. It also provides a phased *framework* for implementation, with different discrete implementation strategies being employed in different phases. REP’s demonstrated effectiveness in promoting uptake of evidence-based practices allows us to focus on its application in varied settings and care models. Use across all projects will allow for further testing and expansion of the framework, particularly through our emphasis on multilevel stakeholder engagement and incorporation of complexity theory.

**Fig. 2:**
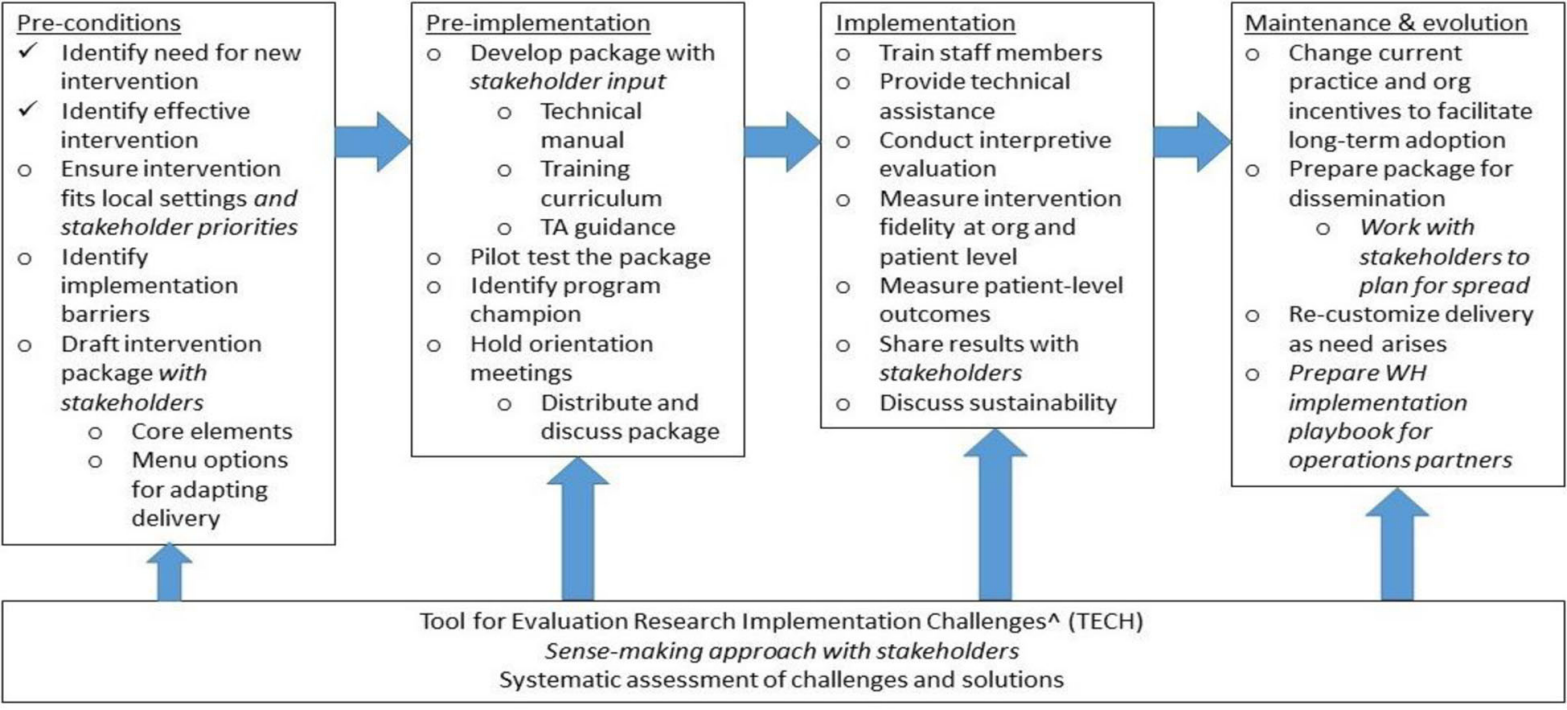
Replicating Effective Programs (REP) Framework (adapted from Kilbourne et al. [17])

The REP framework consists of four phases (Fig. 2): pre-conditions, pre-implementation, implementation, and maintenance/evolution. Careful attention is paid to intervention packaging during pre-conditions and pre-implementation; training, technical assistance, and fidelity assessment during implementation; and recustomizing during maintenance/evolution. During each phase, local context is paramount, with varying deployment of the intervention depending on local priorities, needs, and resources. One of our implementation science goals will be to track the relative importance of each discrete strategy in each phase at each site and across sites, as well as in each project and across projects, to inform user-friendly, implementation practice “playbooks” [16] for our partners.

#### Enhancing REP with multilevel stakeholder engagement and complexity theory

**Fig. 3:**
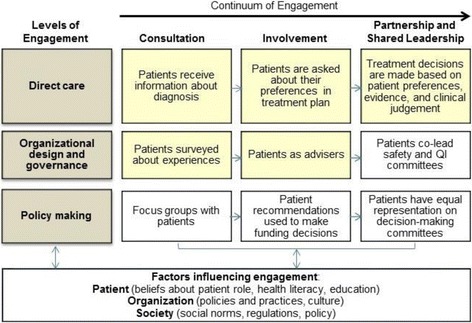
Continuum of Engagement (Adapted from Carman et al. [26]). Key: Yellow boxes addressed in EMPOWER

REP was originally designed to guide dissemination of evidence-based practices in community-based organizations. Kilbourne and colleagues [18] note that “it was not designed to address multilevel barriers to implementation,” so they enhanced REP with facilitation, using implementation experts as external facilitators to provide guidance for overcoming implementation barriers. Interestingly, outcomes were favorable for enhanced REP for their primary implementation outcome of uptake (i.e., completed contacts with Veterans with serious mental illness who had been lost to care), but not for increased utilization of services by patients who had dropped out of care [19]. This prompted us to consider alternate REP enhancements that are (1) more focused on participatory action [20] within complex adaptive systems [21] in VA WH clinical settings [22] and (2) potentially more effective in increasing patient engagement. Accordingly, we draw on complexity theory and multilevel stakeholder engagement (Fig. 3).

#### Complexity theory

Complexity theory postulates that outcomes in complex adaptive systems are nonlinear and unpredictable; it is a highly relational theory, examining how multiple agents involved in implementation interact in complex ways and “make sense” of implementation in different ways [23]. This emphasis on incorporating multiple perspectives and differing priorities is consistent with our VA WH roadmap to delivering gender-sensitive comprehensive care for women Veterans [24], which suggests that multilevel stakeholder engagement is key to improving women Veterans’ health care.

We will operationalize complexity theory through the use of the Tool for Evaluating Research Implementation Challenges (TECH) [25], which was designed to systematically assess impacts of implementation challenges and guide potential solutions. Simpson and colleagues recommend this tool under three pre-conditions: (1) “implementation adaptations are expected due to the emergent nature of complex research settings;” (2) the research environment needs to encourage “spontaneous emergent solutions” and creativity; and (3) all research team members must be empowered to participate. Our approach, where all stakeholders are integral to achieving the impact goal, supports these pre-conditions. TECH, which has been used successfully in VA and community studies, is codified into a series of interactive steps: identifying challenges (e.g., through observing day-to-day dynamics, listening to complaints, asking questions), interpreting the challenges in weekly meetings, generating, and testing solution strategies and, if necessary, addressing regulatory issues. Solution strategies are developed through an open dialog among the team members as well as others who might have perspectives on potential solutions. Although applicable to many different types of research, TECH is especially useful for research in complex adaptive systems as a tool for addressing unexpected challenges in systematic and collaborative ways.

#### Multilevel stakeholder engagement

Carman and colleagues suggest that greater impacts can be achieved by “implementing interventions across multiple levels of engagement,” with specific attention to factors that influence engagement [26]. Their framework conceptualizes an engagement continuum at each of three levels: direct care, organizational design and governance, and policy making. The EMPOWER QUERI focuses on the direct care and organizational levels of engagement (Fig. 3, yellow boxes). At the organizational level, we examine how organizational capacity for innovation impacts implementation of care models designed to promote engagement and retention in care. To guide this process, our implementation strategy features multilevel stakeholder engagement—including patients—consistent with the continuum’s “Involvement” segment. All three projects in the EMPOWER QUERI engage patients in direct care through the entire continuum, as they actively elicit and prioritize clinical actions based on patient preferences. This is consistent with Grande et al.’s “information plus activation plus collaboration” category of patient engagement methods [27].

### Site selection with the women’s health Practice-Based Research Network

EMPOWER has the distinct advantage of being buttressed by the VA HSR&D Women’s Health Research Network (WHRN), which is comprised of the Women’s Health Research Consortium and the Women’s Health Practice-Based Research Network (PBRN), and a research aim on multilevel stakeholder engagement. The VA’s Women’s Health PBRN provides a research infrastructure for investigators seeking to increase inclusion of women in VA research or conduct multi-site women’s focused research in VA. Comprised of 60 VAMCs that together see one-third of women Veteran VA users, the PBRN helps investigators overcome the challenges of multi-site studies through engagement of site leads who have established working relationships with local clinicians and facility leadership. Site leads receive implementation training and have cross-site relationships developed through regular national calls and technical support organized by the PBRN Coordinating Center in Palo Alto. EMPOWER was developed with consistent input from the PBRN site leads (some of whom are on the team), and the projects will all be conducted at PBRN sites.

### EMPOWER QUERI measures

To take advantage of an integrated set of implementation projects coalescing under one impact goal and being driven by one implementation strategy, we will use a common set of measures across all projects according to the REP phases (Table 1). This approach will provide us with a master data set for the core measures, allowing for analyses across projects. For implementation measures, we will use a cross-QUERI evaluation and analysis approach, with tailoring to each project’s specific aims. Each project will necessarily include additional project-specific measures, described within the body of each project description. The DPP QI project data collection and analysis plan are tightly aligned with its non-research operations aims, thus only those data required to accomplish its aims will be collected and analyzed as part of the QI project. During summative analysis, the EMPOWER QUERI research teams will request IRB approval to augment or combine existing VA data sources, including data collected by the DPP project as QI, with other data, to advance the program’s overall aims.

**Table 1:** Evaluation activities and measures by Replicating Effective Programs (REP) Phase

Pre-conditions	Pre-implementation	Implementation	Maintenance and evolution
• TECH • Field notes • Key stakeholder* interviews • ICS, ILS, ICBS, PCIS, MORE • Patient qualitative interviews	• TECH • Field notes	• TECH • Field notes • Key stakeholder interviews • Patient interviews	• TECH • Field notes • Key stakeholder interviews
		Impact-focused evaluation	
		• Referral monitoring • Engagement • Retention • Patient measures	

Consistent with the engagement framework, we will examine organizational, provider, and patient factors that influence engagement, as well as core structural features (e.g., configuration of WH care, number of women Veterans served, number of designated WH providers, size of patient panels, and Patient-Aligned Care Team (PACT) structures).

#### Organizational climate and readiness factors

We will focus on brief, pragmatic measures of climate and readiness for implementation. The *Implementation Climate Scale* (*ICS*) [28] is an 18-item measure of the local strategic climate for implementation, capturing six dimensions of organizational context that reflect employees’ ratings of the extent to which their organization prioritizes and values successful implementation of evidence-based practices. The *Implementation Leadership Scale* (*ILS*) [29] is a 12-item scale of clinic-level leadership for implementation. The *Implementation Citizenship Behavior Scale* (*ICBS*) [30] is a 6-item measure that captures critical behaviors employees perform to go above and beyond the call of duty to support implementation, including helping other employees on implementation-related activities and keeping informed about issues related to evidence-based practice and implementation efforts. The *Measure of Organizational Readiness for Patient Engagement* (*MORE*) [31] is a 33-item survey that assesses organizational willingness and ability to effectively implement patient engagement in healthcare. Permissions for use have already been obtained.

#### Provider factors

The *Perceived Characteristics of Intervention Scale* (*PCIS*) [32], a 20-item assessment, was developed and tested among VA healthcare providers to assess their views of interventions. Based on constructs from Diffusion of Innovation theory, the PCIS is a reliable measure of perceived characteristics of interventions, with preliminary support for its validity.

#### Patient factors

For our impact evaluation, patient-level measures across projects will assess patient activation, experiences of care, social support, health-related quality of life, mental health symptoms (anxiety, depression), and CV risk. Patient engagement and retention will be assessed specifically in each project per the parameters of their respective interventions. Patient activation will be assessed using a 6-item version of the *Patient Activation Measure* (*PAM*) [33, 34]. PAM items are rated on a 4-point Likert scale ranging from 1 (strongly agree) to 4 (strongly disagree). The measure yields an activation score from 0 (least activated) to 100 (most activated), which is transformed into an ordinal indicator of four levels of activation ranging from level 1 (least) to level 4 (most activated). Items from the *Consumer Assessment of Healthcare Providers and Systems* (*CAHPS*) *Patient-Centered Medical Home* (*PCMH*) *Survey* [35] will be used to measure patient experience with care (e.g., communication). The short-form *Interpersonal Support Evaluation List* (*ISEL*) [36, 37] is a widely used 12-item instrument that assesses perceptions of social support. The ISEL has been subjected to extensive psychometric testing and has shown to be internally consistent and valid [38]. Health-related quality of life will be measured using the *Healthy Days* measure [39]. The measure consists of a four-item core module assessing health status, four items measuring activity limitation, five symptom-related items that assess the days in the past month the respondent experienced symptoms related to pain, depression, anxiety, and sleep problems, and one vitality item. The five-item *Overall Anxiety Severity and Impairment Scale* (*OASIS*) [40] will be used to assess anxiety. OASIS scores demonstrate robust correlations with global and disorder-specific measures of anxiety, and this scale is the only available measure that captures severity and impairment *across anxiety disorders* [41]. The *Patient Health Questionnaire* (*PHQ*) is the depression module of the PRIME-MD diagnostic instrument, which scores each of the nine DSM-IV criteria as “0” (not at all) to “3” (nearly every day). The PHQ-9 is also a reliable and valid measure of depression severity [42]. To assess *cardiovascular risk*, we will use items from the CV project patient worksheet regarding traditional CV risk factors, family history of CV disease, pregnancy and gestational history, and smoking status.

### Data collection

#### Qualitative data collection

Semi-structured qualitative interviews will be conducted during the pre-conditions, implementation, and maintenance phases with non-patient key stakeholders (defined in each project) (*n* ~ 15 per site). Baseline interviews will examine usual care for the relevant care condition; knowledge, attitudes, and behaviors regarding the specific care model; and anticipated barriers and facilitators to implementation. The mid-implementation and final follow-up interviews will assess the following: (1) usual care vs. new care model (relative advantages, complexity, etc.), (2) barriers and facilitators to implementation [43], and (3) perceptions of acceptability and feasibility of the care model. In the REP implementation phase, semi-structured interviews will be conducted with a subsample of patients at baseline and 6-months post baseline to assess perceptions of acceptability of and satisfaction with the care model to which they were exposed. Ethnographic field notes will be taken by research team members *throughout implementation* to capture aspects of the context of implementation and otherwise unmeasured aspects of usual care. Also, minutes will be recorded for all project meetings (including trainings) and conference calls. In addition, substantive emails and other communications regarding implementation will be archived and analyzed.

#### Quantitative data collection

Key stakeholders will be asked to complete written questionnaires during or near the same time as the qualitative interviews. We also administer baseline and post-implementation surveys to patients, as described in detail in individual project narratives (see Additional files 1, 2, and 3). The 1-year project focused on diabetes prevention will collect DPP session/module participation and weight change for participants. The two multi-site, multi-year implementation research projects will utilize VA medical record data from the VA Corporate Data Warehouse for patient demographics, vitals and lab results, BMI, mental health diagnoses, comorbidities, and service utilization as well as tracking implementation. For example, for the CV Toolkit project, measures of provider template use (percent of patients for whom the provider used the Computerized Patient Record System (CPRS) template) and number of CV-related referrals (total count of CV-related referrals, *Gateway to Healthy Living-*specific referrals and other services) will be captured from CDW. For CCWV, we will also track referrals to the CCWV care manager. For each referral, we will create a dichotomous variable (1 = referral made) to create a count measure of total referrals. Further details can be found in Additional file 1.

### Data analysis plan

#### Qualitative analysis

All semi-structured interviews will be digitally recorded and transcribed verbatim. Transcripts will be reviewed, edited for accuracy, and summarized by members of the research team. Consistent with our team’s approach across multiple projects, matrix analysis methods [44] will be used for rapid turn-around of the results [45] to share with our Strategic Advisory Group. In-depth analysis of the qualitative data will be conducted using ATLAS.ti, a qualitative data analysis software program that allows for fluid interaction of data across types and sources. Initially, a top-level codebook will be developed for the baseline interviews based on the semi-structured interview guide [46]. Using a constant comparison analytic approach, this codebook will be elaborated upon based on emergent themes, and it will be adjusted as each round of interviews is reviewed. Interviews will be compared within each clinic, across clinics, and over time. Additional sources of qualitative data (i.e. meeting minutes, field notes, and archival information) will also be included in the data set and will be coded separately and in relation to the interview data. These multiple approaches and groupings are easily facilitated within the software program, which has the capacity to group data in multiple ways and which allows the qualitative researchers maximum flexibility in negotiating a complex narrative dataset.

In the pre-conditions transcripts, we will identify commonly shared knowledge, attitudes, and beliefs related to care model structures, processes, and effectiveness and the potential for effectiveness. We will synthesize this information with survey data to create baseline summaries of care as usual and to tailor our marketing and implementation strategies for use at the sites. In mid-implementation interview data, we will identify factors facilitating and impeding implementation of the care model, and strengths and weaknesses of the model as implemented. We will assess the extent to which components of the model are being implemented and which components are efficient and easy to incorporate into routine care. We will explore whether particular components appear to be of limited value in improving care and examine clinic and provider characteristics associated with varying levels of care model implementation and effectiveness. In post-implementation interview data, we will take a summative approach to characterizing overall experiences of and perspectives on implementation, with a particular focus on recommendations for scale up and spread [47].

#### Impact-focused evaluation

Secondary aims for each project focus on factors that empower women Veterans to engage in and benefit from care. We will use generalized linear mixed models (GLM) to evaluate (a) cross-sectional relationships of patient activation, health-related quality of life, and care experiences at enrollment, with provider and site characteristics, adjusting for patient social and demographic characteristics and (b) prospective changes in patient activation, health-related quality of life, and care experiences from enrollment to 6-month follow-up, adjusting for patient, provider, and site characteristics. We will also construct multilevel mediation and moderation models to explore whether engagement-related factors such as patient activation, strength of treatment preferences, or the communication subscale of the CAHPS are associated with greater benefit from or satisfaction with care.

#### Non-randomized stepped wedge for implementation trials

The two implementation research studies described in this protocol have been registered as follows: Facilitating Cardiovascular Risk Screening and Risk Reduction in Women Veterans: Trial registration NCT02991534, registered 9 December 2016; Implementation of Tailored Collaborative Care for Women Veterans: Trial registration NCT02950961, registered 21 October 2016.

Both CV Toolkit and CCWV implementation studies will use stepped wedge designs, which rely on sequential roll-out to participating sites over time, while using other sites as controls until they begin implementation [48]. Consistent with our substantial prior experience using these designs in VA and armed with complexity theory’s recognition of nonlinearity of implementation [49], we will use *a non-randomized* stepped wedge design (rather than randomized) given their suitability for studying implementation. This design acknowledges that sites are heterogeneous, face multiple constraints and, as a result, are ready to adopt interventions at different rates. The non-randomized design explicitly considers the timing of implementation spread and addresses the statistical issues introduced by lack of randomization in implementation starts and processes. We will analytically compensate for the design by collecting patient-, provider-, and site-level data that may be associated with timing of the adoption of each intervention.

**Fig. 4:**
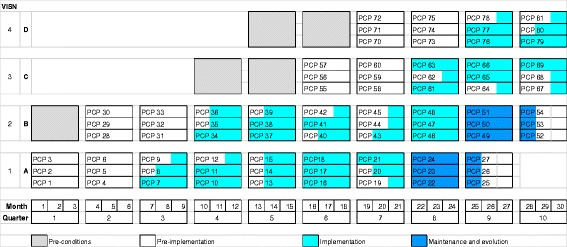
EMPOWER QUERI Non-randomized Stepped Wedge Design

Non-randomized stepped wedge designs make efficient use of all data available for within-site and between-site comparisons. For the within-site comparison, sites act as their own controls in an evaluation that compares sites pre versus post implementation. The comparison examines sites as they cross-over from control to intervention states. The between-site comparison evaluates the intervention period for a site vs. all other intervention and control periods for all sites. By having these two types of comparisons, the design improves on the validity of the evaluation of the intervention, by accounting for historical time trends that may occur outside of the intervention and for site contextual characteristics that may affect site performance. We will include three levels in our hierarchical non-randomized stepped wedge models: (1) patient, (2) time of intervention (i.e., when a primary care provider starts using the intervention), and (3) site. Outcomes of interest are measured for all patients at each site within the given intervention time period. We will include four PBRN sites in each of our two implementation trials. Figure 4 provides an example of the multilevel nature of site and provider adoption over time and by REP phase (e.g., pre-conditions for 6 months, pre-implementation for 6 months, implementation for 15 months, and maintenance/evolution for 4 months). Data collection will occur in each of these phases. Similar to previous non-randomized stepped wedge studies, implementation initiates with the primary care providers (PCPs). Figure 4 depicts site A initiating at quarter 3, site B at quarter 4, and so on, with three PCPs at each site, each quarter, for a total of 81 PCPs. The PCPs “turn on” (i.e., use the template in the CV project; refer to the care manager in the CCWV project) as they switch from pre-implementation to implementation.

#### Power analysis

Our goal with the power analysis was to ascertain the number of providers who would need to “turn on” the intervention (i.e., the number of PCPs who use the CV template and the number of referrals to the CCWV care manager), as well as the minimum number of patients that would need to be exposed to the care model in each project. Consistent with our non-randomized stepped wedge design, the power analysis presented here is for a three-level hierarchical linear model where patients are clustered within PCPs within sites. In the final analysis structure, patients are the level 1 units, the timing of PCP template use/referrals represent level 2 units, and sites are the level 3 units. The outcome of interest for CV Toolkit is the number of referrals to services (e.g., *Gateway to Healthy Living* and other services) and for CCWV is the number of patients who completed the care model sessions out of all patients referred. The outcome is binary (referred/retained vs. not) and measured at the patient level (level 1). Treatment effect is measured at level 2, i.e., timing of when the PCPs turn on. Parameters required for calculating power are alpha (*α*) = 0.05, sample size of patients clustered within referring PCPs (*n*) = at least 260 (based on prior work), the number of sites (*K*) = 4, the Plausible for Retention among those with low utilization (PI) = 5 to 85%, and effect size variability (ESV) = medium or 0.05. Based on the resulting power curve, we need at least 8.78 (rounded to 9) referring PCPs per site to achieve power at 0.80. Our power analyses assume that the treatment effect is linear over time, i.e., patients that are retained longer have proportionally better outcomes. While selected details of the resulting dose response curve may vary, sensitivity analyses demonstrate a reasonable approximation enabling detection of treatment effects.

## Discussion

The EMPOWER QUERI is important, timely, and essential to inform policy and programming to ensure women Veterans’ equitable access to high quality health care within VA. Addressing the needs of women Veterans is a key strategic priority for VA and our operation partners, and the funding of this study represents an exciting opportunity to develop tailored, gender-specific care models in close collaboration with administrative, provider, and patient stakeholders. In addition, because this project has been designed and is being conducted in close partnership with VA leadership, we anticipate that, should implementation prove successful, we will have many opportunities to support scale up and spread of the DPP, CV Toolkit, and CCWV care models.

The EMPOWER QUERI faces potential limitations common in implementation research. Engaging sites for participation in implementation of innovative care models may create challenges, given the level of commitment and, in some cases, local re-organization involved. We expect our collaboration with the WH-PBRN and commitment to tailoring projects to meet the needs of sites and stakeholders will facilitate site recruitment. While women Veterans are a rapidly growing but numerical minority among VA users, previous research endeavors have had difficulty with patient recruitment for trials at the local level. Moreover, programs with lifestyle and health behavior change report low participation among women Veteran VA users [50]. These experiences indicate that each of our three projects may experience low rates of engagement unless we are able to capitalize on patient needs and willingness to try new innovations at VA facilities.

Despite these challenges, EMPOWER QUERI offers myriad opportunities to further current developments in implementation science. First, the cross-project use of an enhanced REP framework drawing upon both complexity science and multilevel stakeholder engagement presents a unique opportunity to compare the utility of this framework across multiple sites in facilitating adaptation and implementation of two distinct care models (CV Toolkit and CCWV). Close involvement of stakeholders at multiple levels and across every phase of this project provide a model for tailoring care delivery to meet the needs of women in VA settings, while use of the TECH provides a structured process of creative resolution for the inevitable challenges that emerge. In particular, the formal partnership with a council of women Veterans across projects and the incorporation of a female Veteran peer to lead DPP interventions are novel examples of patient-partnerships to help shape QI and research objectives within VA. The use of non-randomized stepped wedge analysis offers an innovative strategy for evaluating implementation effectiveness in real-world settings, enhancing the validity of findings by allowing for examination of both within- and cross-site impacts. The implementation playbooks to be developed for operations partners will build upon study findings and stakeholder input to establish an implementation blueprint for future spread that is both formal in recommending use of specific strategies and flexible in acknowledging the differing needs of sites with varied structures and resources. And finally, the direct examination of patient engagement as a mediator in implementation and intervention outcomes addresses the critical role of the patient in implementation success, which has too often been a gap in prior research [51]. Taken in sum, EMPOWER aims to advance understandings of theory, methods, and mechanisms in implementation research and to bring the accumulated knowledge to bear in rapidly advancing healthcare delivery for women Veterans.

## Additional files

Additional file 1: Individual project description: Tailoring VA’s Diabetes Prevention Program to Women Veterans’ Needs (Tannaz Moin (PI) and Sally Haskell (Co-PI)). (DOCX 48 kb)

Additional file 2: Individual project description: Facilitating Cardiovascular Risk Screening and Risk Reduction in Women Veterans (Bevanne Bean-Mayberry (PI) and Melissa Farmer (Co-PI)). (DOCX 61 kb)

Additional file 3: Implementation of Tailored Collaborative Care for Women Veterans (Alison Hamilton (PI) and Ariel Lang (Co-PI)). (DOCX 50 kb)

## Abbreviations

CCWV: Collaborative Care for Women Veterans
CPRS: Computerized Patient Record System
CV: Cardiovascular
EMPOWER: Enhancing Mental and Physical health of Women through Engagement and Retention
MH: Mental Health
NCP: National Center for Health Promotion and Disease Prevention
OPCC&CT: Office of Patient Centered Care and Cultural Transformation
PACT: Patient-Aligned Care Teams
PC-MHI: Primary Care-Mental Health Integration
PBRN: Practice-based research network
PCP: Primary care provider
QUERI: Quality Enhancement Research Initiative
REP: Replicating Effective Programs
TECH: Tool for Evaluating Research Implementation Challenges
WH: Women’s Health
WHRN: Women’s Health Research Network
WHS: Women’s Health Services
