# Supplementary material for: Enhancing Mental and Physical Health of Women through Engagement and Retention (EMPOWER): a protocol for a program of research
Source: Implement Sci. 2017 Nov 7;12:127. doi: 10.1186/s13012-017-0658-9 (PMC5678767; doi:10.1186/s13012-017-0658-9)
Supplement: Supplementary file 1 — Individual project description: Tailoring VA’s Diabetes Prevention Program to Women Veterans’ Needs (Tannaz Moin (PI) and Sally Haskell (Co-PI)). (DOCX 48 kb) [file 13012_2017_658_MOESM1_ESM.docx]

**Project 1: Tailoring VA’s Diabetes Prevention Program To Women Veterans’ Needs**

*Specific Aims*

Type 2 diabetes is a common disease, affecting 25% of Veterans, and is associated with significant morbidity and mortality. However, type 2 diabetes may be prevented using evidence-based diabetes prevention services that target high-risk patients with pre-diabetes. Pre-diabetes is common, with about one in three adults aged 20-64 years and one in two aged 65 years or older having the condition [1], which is associated with an increased risk of type 2 diabetes and well-known diabetic complications, such as retinopathy [2]. Studies have also demonstrated that CV disease risk has a linear association with glycemia well below diabetes’ diagnostic threshold [3]. Women with prediabetes are at increased risk as up to 30% may progress to diabetes over 3-4 short years [4]. Persistent gender disparities in VA quality of care for diabetes and CV risk factors require innovative approaches to target and promote diabetes prevention among women Veterans.

VA Greater Los Angeles (GLA) was one of four national sites participating in VA’s Diabetes Prevention Program (VA DPP) in close partnership with the VA National Center for Health Promotion & Disease Prevention (NCP) and the Diabetes QUERI. Originally designed to rely on in-person professional lifestyle coaches with master’s-level training in nutrition/dietetics, the VA DPP was subsequently expanded to include an online component in response to (a) difficulty recruiting for in-person programs which require frequent face-to-face meetings and (b) evidence supporting effectiveness of online DPP in non-VA settings. Use of peers, or lay community members, has also been shown to be as effective as DPP interventions delivered by professional coaches in non-VA settings [5–11]. Furthermore, peer-led and online DPP delivery models have been associated with lower cost and resource requirements, making them more amenable to broad scale-up [12]. Given gaps in data on gender differences in program response, we conducted qualitative interviews with women veteran participants in our ongoing multi-site online DPP program and found high levels of engagement, retention, and clinically meaningful weight loss over 16 weeks of participation in online DPP [13]. Our growing interest in peer-led DPP interventions led to funding of a mixed gender pilot program to further inform our QI work.

Tailoring care to the needs of women veterans is a top priority expressed by all of our Program partners, as well as being central to the VA’s WH services research and QI agendas [14,15]. A growing body of literature has identified the importance of women-centered service delivery to reduce disparities in care and improve women veterans’ experiences and outcomes of VA care. For example, Bastian and colleagues have shown that women veterans’ experiences with VA outpatient care are better when seen by designated women health providers [16]. Studies have shown that women veterans are more reluctant to regularly attend in-person VA-sponsored programs due to the nature of the predominantly male environment [17]. They may also feel uncomfortable discussing weight and exercise in groups that include face-to-face interaction with men [18]. A recent review revealed gender differences in response to, and preferences for, various weight loss programs [19]. Therefore, the use of women-focused groups will allow us to better align our DPP interventions with women veterans’ preferences and needs, which is consistent with our own anecdotal experiences over past three years of DPP delivery at GLA. We anticipate that women veterans will have greater comfort and identification with peers who are also women veterans with pre-diabetes (as opposed to male veterans or non-veterans). Our work has also shown that online DPP interventions are positively viewed by women veteran participants. As such, we propose a six-month pilot to implement and evaluate peer-led and online DPP interventions tailored for women veterans at VA GLA. Implementation outcomes include adoption, acceptability, feasibility, and satisfaction. Patient-level outcomes include activation, engagement, and retention. Our specific aims are to:

1. Leverage existing women’s health and DPP infrastructure at GLA to implement a six-month tailored DPP intervention for 40 women veterans with pre-diabetes, allowing participants to choose either a peer-led, in-person or an online DPP intervention, and,
2. Use mixed methods to: a) document the implementation process; b) identify barriers and facilitators to adoption, acceptability, feasibility, and satisfaction; and c) assess women veterans’ activation as well as engagement and retention in the tailored DPP intervention.

*Rationale*

Diabetes prevention and DPP interventions tailored to women veterans’ needs are critical in VA. Over the past 20 years, several large-scale, randomized studies, including the Diabetes Prevention Program (DPP) study, have shown that lifestyle interventions result in a 58% reduction in diabetes incidence [2,20]. The need for real-world adaptations of the DPP that optimize effectiveness, minimize costs, and improve sustainability is evident across all healthcare settings [21]. Within VA, NCP has prioritized the goal of providing veterans at risk for diabetes access to evidence- based lifestyle interventions. GLA has closely partnered with NCP and the Diabetes QUERI on a multi-site VA DPP Clinical Demonstration Project since 2012 to help accomplish this goal. Our results, though not yet published, have already contributed to the national re-design of the mixed-gender VA MOVE! Program. However, studies have shown that women veterans are more reluctant to regularly attend VA programs in-person due to the predominantly male environment [17]. Robertson and colleagues have also shown gender differences in response to, and preferences for, various weight loss programs [19]. Therefore, the use of women-only groups may allow us to better align lifestyle interventions, such as DPP, with women veterans’ unique needs and preferences.

Both peer-led and online DPP delivery models have been associated with improved health outcomes and weight loss as compared to interventions delivered by health or medical professionals [11]. A peer-led DPP delivery model is patient-centered in many ways, including; 1) leadership and skill building among veteran peers who will help deliver the main intervention, 2) the opportunity to enhance engagement in the intervention through connections that occur naturally among participants and peers (i.e., prior military experience or common diagnosis of prediabetes), and 3) the opportunity to enhance participation by providing relevant examples of success through peers who have already achieved desired lifestyle changes. The incorporation of peers in VHA healthcare is also a growing area of interest with several successful examples of peer models in VA mental health initiatives [22,23]. Additionally, Heisler and colleagues also demonstrated that peer support programs are an effective means of managing chronic illnesses, such as diabetes, among veterans [24,25].

Since women often cite competing demands as a significant barrier to lifestyle intervention adherence [26,27], we realize regular attendance of an in-person DPP intervention may be challenging for some women Veterans with competing demands. For some women veterans, distance may also be a barrier because up to one-third are estimated to live in rural or highly rural areas [28]. Online DPP interventions have the potential to reduce or eliminate many of these potential barriers, since they are delivered asynchronously and easily accessed, affording women veterans greater convenience and flexibility. Online interventions have been shown to improve behavioral outcomes including increased exercise time, increased knowledge of nutritional status, and 18-month weight loss maintenance [29]. Our work has shown that women veterans’ early experiences with the online DPP intervention *Prevent,* currently in use at GLA and three other VAMCs, was positive and that women veterans viewed this intervention as an appealing way of initiating lifestyle changes that made them feel accountable in achieving their daily goals [13].

*Procedures*

Project Design. This is a one-year quality improvement project based at VA GLA. To achieve Aim 1, we will leverage the team’s prior VA DPP experience and the existing screening, referral and follow-up processes to tailor the program to women veterans’ needs.

Implementation strategy. Aligned with the EMPOWER QUERI level aims, we will use the REP implementation strategy and multilevel stakeholder engagement. Key partners include the GLA WH Medical Director and Women Veteran Program Manager (WVPM), both of whom are members of our Strategic Advisory Group. Given immense local experience in DPP delivery, we have already successfully completed many of the REP phases, including pre-conditions, pre-implementation, and training of staff members, which is part of the implementation phase.

Implementation Phase: We will invite eligible women veterans to participate in a women-focused DPP intervention that best aligns with their needs and preferences, either an in-person, peer-led or online intervention. As we have done for the past three years, all eligible participants initially receive a DPP invitation letter from their PCP. Women veterans who do not opt-out of participating will receive a call from qualified staff to describe the DPP interventions in detail, answer questions, review their needs and preferences (e.g., competing demands) and confirm their choice of either the in-person or online DPP. For the in-person DPP intervention, existing VA DPP professional coaches will supervise our existing woman veteran peer coaches who will lead in-person DPP sessions. For the online DPP option, we will adapt our existing online DPP intervention to provide women-focused online groups with certified female coaches. Both interventions will enroll on a rolling basis and are described in more detail below. During the implementation, the PIs and team will meet biweekly with the WH Medical Director, WVPM, professional and peer DPP coaches, and other relevant providers to address any implementation challenges, using TECH to reach consensus-based solutions. Also, the project RA has been trained to take field notes regarding implementation processes and observations about feasibility and acceptability.

Peer-led, in-person DPP intervention: Our in-person DPP program will continue to include the Group Lifestyle Balance (GLB) curriculum, shown to be effective in a variety of clinical and non-clinical settings [30,31]. Based on the original DPP curriculum, GLB consists of 16 core sessions over six months. The sessions focus on healthy eating, caloric and fat intake, incorporation of moderate physical activity. Preliminary results from the VA DPP Clinical Demonstration Project support the feasibility and acceptability of this rigorous curriculum, which has been very well received by our veterans. Heisler’s key elements for success of peer-led, group self-management classes, such the use of one dedicated staff member to coordinate logistics (i.e., an RA), the continued opportunity for exchange and skill development (i.e., weekly meetings with professional coaches), and a curriculum formatted on behavioral approaches found to be effective (i.e., GLB), have already been incorporated [25]. Salary support is also listed as a key element of success and will primarily account for the bulk of the project budget [25].

Existing VA DPP professional coaches will help train, support and supervise our woman veteran peer DPP coaches. Our female veteran peer coach was a graduate of our prior in-person VA DPP programs who has successfully achieved all the recommended lifestyle changes, leading to significant weight loss and lowering of her diabetes risk. Our female veteran peer coach has attended daily training sessions, including detailed review of the VA DPP curriculum and role-playing of each DPP session, where she receives feedback from our professional coaches. Our peer training program is extensive and provides a comprehensive, standardized overview of the curriculum and core concepts and training to address minimum core competencies in organizational skills, communication (i.e., active listening and nonjudgmental communication), support /empathy, engagement, group leadership, professionalism, and patient confidentiality/HIPAA. Our peer coach will have several months of actual DPP teaching experience by the anticipated start date of this proposal. Ongoing supervision will occur during class sessions and in the form of bi-weekly meetings between our peer and professional coach to address participant progress and issues that arise.

Online DPP intervention**:** Our VA DPP work included over two years of experience with a commercially-available online DPP-based group lifestyle intervention, known as *Prevent,* developed by Omada Health. *Prevent* integrates educational modules, health coaching, and tracking tools [32]. A unique feature of this program is that it leverages social media principles to deliver virtual DPP in a small group format. *Prevent* has been shown to meet the Centers for Disease Control and Prevention (CDC) Diabetes Prevention and Recognition Program (DPRP) outcome standards [33], and weight loss outcomes of other DPP translations [32]. For the purposes of this pilot QI project, we will enroll eligible women veterans who chose the online option in women-only groups that include certified female coaches. All participants will be provided with a six-month membership to *Prevent* consisting of 16 weekly modules of intensive core curriculum followed by a two-month maintenance phase (two “post-core” monthly modules). Core modules are released each week and participants choose when to log in and for how long. A certified professional female health coach will be assigned to each group to help deliver the curriculum, answer questions, and monitor group interactions in real time to ensure an appropriate and positive virtual group environment. Participants can post messages to the entire group or send private messages to the health coach.

DPP participants: To ensure successful study completion within the limited time frame of this proposal, we plan to enroll up to 40 women veterans on a rolling basis. Women GLA VA patients with a diagnosis of prediabetes based on either an HbA1c (5.7-6.5%) or fasting blood glucose (100-125 mg/dL) in the past 12 months will be eligible to participate. Main exclusion criteria will include a history of diabetes, chronic use of medication known to substantially affect glucose metabolism, history of bariatric surgery, current pregnancy or lactation, active malignancy, a diagnosis of dementia, and any contraindications to the gradual adoption of moderate physical activity (i.e., recent cardiovascular event in past six months). Participants will be recruited from outpatient WH Clinics at the West Los Angeles and Sepulveda Campuses and will need documentation of routinely required medical clearance to participate in moderate physical activity from a VA physician. The first DPP groups will begin as soon as eight eligible women veterans are identified.

*Implementation Evaluation*

We will use mixed methods to evaluate implementation.

Qualitative data collection: We will collect the EMPOWER QUERI Program patient measures from all participants. Semi-structured interviews will be conducted when the DPP interventions begin (“baseline”) and repeated at six months post-baseline to inform broader implementation and the EMPOWER QUERI implementation projects. At pre-implementation, approximately five key stakeholders (peer/professional coaches, WH providers) and 10 DPP enrollees will be interviewed. At post-implementation about five staff, eight DPP graduates and eight DPP non-completers will be interviewed. To the extent possible, the same individuals will be interviewed at pre and post, though additional patients will be added to the post-implementation sample in order to investigate DPP completion versus drop-out. All interviews will be conducted in-person in the women’s clinic and will last approximately 30 minutes. The interview questions will span attitudes toward the intervention (e.g., satisfaction), observations of effectiveness and acceptability, barriers to and facilitators of implementation, and anticipated sustainability. In interviews with non-patient key stakeholders, we will particularly examine Diffusion of Innovation [34] constructs that are likely to influence the acceptability, adoption and sustainability of peer and online DPP interventions, such as: perception of the relative advantage of the DPP delivery model over other available alternatives (including MOVE! which is usual care in the VA) and complexity.

Qualitative analysis: We will summarize each interview according to a defined set of interview domains, and will examine similarities and differences across types of participants [35]. Code development will be driven by the summaries. Coding will be conducted using ATLAS.ti. The two waves of interviews will be examined for possible changes in attitudes, barriers and facilitators over time.

In addition to Implementation Core activities, we will compare in-person and online DPP care models in terms of a) satisfaction; b) activation (preferred choice); c) engagement (minimum nine or more sessions completed, in accordance with CDC guidelines); and d) retention (rates of completion of all 16 sessions).

Quantitative Data Collection and Analysis**:** We will analyze EMPOWER QUERI program common measures on all participants, including demographics, patient activation, experiences of and satisfaction with care, health-related quality of life, anxiety and depression screens, and cardiovascular risk screen using self-administered surveys. As guided by CDC national standards for DPP delivery, we will collect data on program attendance (module completion for the online DPP intervention) and documentation of physical activity minutes [33]. As noted above, the CDC national guidelines require that DPP participants complete a minimum of nine sessions [33], so nine or more sessions can be considered as a minimum threshold for program engagement. We will also assess trends in the main effects of the DPP modality chosen on percent weight loss at 16 weeks and six months for DPP participants. We will stratify results according to enrollment status and use descriptive analyses to compare percent weight loss, self-reported physical activity, engagement and retention between the in-person and online DPP options. Chi-squared and t-tests will be used to test differences (P < .05) between two DPP options but given the small number of patients we do not expect to see statistically significant differences. All analyses will be done using SAS version 9.3 (SAS Institute Inc., North Carolina) and STATA, version 12.1 (STATACorp, Texas).

*Impact*

The knowledge gained from this QI project will inform the two larger implementation projects in the EMPOWER QUERI Program, as well as future diabetes prevention efforts in the VA. This work will allow us to characterize the impact of gender-specific interventions and peers on primary prevention and risk reduction services in the VA, as well as preferences regarding modalities of program delivery (in-person vs. online). This proposal aims to improve the quality of diabetes prevention services for women veterans through a patient-centered, proactive, personalized care model focused on the unique needs and preferences of women veterans, while leveraging several years of ongoing DPP experience at GLA.

**References**

1. CDC. National diabetes fact sheet: national estimates and general information on diabetes and prediabetes in the United States. Atlanta, GA: .S. Department of Health and Human Services, Centers for Disease Control and Prevention; 2011.

2. Reduction in the Incidence of Type 2 Diabetes with Lifestyle Intervention or Metformin. N. Engl. J. Med. 2002;346:393–403.

3. Endocrinologists ACoEatAAoC. Consensus Statement. 2008.

4. Nichols GA, Hillier TA, Brown JB. Progression From Newly Acquired Impaired Fasting Glusose to Type 2 Diabetes. Diabetes Care. 2007;30:228–33.

5. Frayne S, Phibbs C, Saechao F, et al. Sourcebook: Women Veterans in the Veterans Health Administration. Volume 3. Sociodemographics, Utilization, Costs of Care, and Health Profile. Washington DC: Women’s Health Services, Veterans Health Administration, Department of Veterans Affairs; 2014 Feb.

6. Whitehead AM, Czarnogorski M, Wright SM, Hayes PM, Haskell SG. Improving Trends in Gender Disparities in the Department of Veterans Affairs: 2008–2013. Am. J. Public Health. 2014;104:S529–S531.

7. Goldstein KM, Melnyk SD, Zullig LL, Stechuchak KM, Oddone E, Bastian LA, et al. Heart Matters: Gender and Racial Differences Cardiovascular Disease Risk Factor Control Among Veterans. Womens Health Issues. 2014;24:477–83.

8. Vimalananda VG, Biggs ML, Rosenzweig JL, Carnethon MR, Meigs JB, Thacker EL, et al. The influence of sex on cardiovascular outcomes associated with diabetes among older black and white adults. J. Diabetes Complications. 2014;28:316–22.

9. Runnals JJ, Garovoy N, McCutcheon SJ, Robbins AT, Mann-Wrobel MC, Elliott A, et al. Systematic Review of Women Veterans’ Mental Health. Womens Health Issues. 2014;24:485–502.

10. Hamilton AB, Frayne SM, Cordasco KM, Washington DL. Factors Related to Attrition from VA Healthcare Use: Findings from the National Survey of Women Veterans. J. Gen. Intern. Med. 2013;28:510–6.

11. Ali MK, Echouffo-Tcheugui JB, Williamson DF. How Effective Were Lifestyle Interventions In Real-World Settings That Were Modeled On The Diabetes Prevention Program? Health Aff. (Millwood). 2012;31:67–75.

12. Lawlor MS, Blackwell CS, Isom SP, Katula JA, Vitolins MZ, Morgan TM, et al. Cost of a Group Translation of the Diabetes Prevention Program. Am. J. Prev. Med. 2013;44:S381–S389.

13. Moin T, Ertl K, Schneider J, Vasti E, Makki F, Richardson C, et al. Women Veterans’ Experience With a Web-Based Diabetes Prevention Program: A Qualitative Study to Inform Future Practice. J. Med. Internet Res. 2015;17:e127.

14. Yano EM, Bastian LA, Bean-Mayberry B, Eisen S, Frayne S, Hayes P, et al. Using Research to Transform Care for Women Veterans: Advancing the Research Agenda and Enhancing Research–Clinical Partnerships. Womens Health Issues. 2011;21:S73–S83.

15. Yano EM, Hayes P, Wright S, Schnurr PP, Lipson L, Bean-Mayberry B, et al. Integration of Women Veterans into VA Quality Improvement Research Efforts: What Researchers Need to Know. J. Gen. Intern. Med. 2010;25:56–61.

16. Bastian LA, Trentalange M, Murphy TE, Brandt C, Bean-Mayberry B, Maisel NC, et al. Association between Women Veterans’ Experiences with VA Outpatient Health Care and Designation as a Women’s Health Provider in Primary Care Clinics. Womens Health Issues. 2014;24:605–12.

17. Fontana A, Rosenheck R. Treatment of Female Veterans with Posttraumatic Stress Disorder: The Role of Comfort in a Predominantly Male Environment. Psychiatr. Q. 2006;77:55–67.

18. Rossiter AG, Smith S. The invisible wounds of war: Caring for women veterans who have experienced military sexual trauma: The invisible wounds of war. J. Am. Assoc. Nurse Pract. 2014;26:364–9.

19. Robertson C, Avenell A, Boachie C, Stewart F, Archibald D, Douglas F, et al. Should weight loss and maintenance programmes be designed differently for men? A systematic review of long-term randomised controlled trials presenting data for men and women: The ROMEO project. Obes. Res. Clin. Pract. 2016;10:70–84.

20. Lindström J, Louheranta A, Mannelin M, Rastas M, Salminen V, Eriksson J, et al. The Finnish Diabetes Prevention Study (DPS): Lifestyle intervention and 3-year results on diet and physical activity. Diabetes Care. 2003;26:3230–6.

21. Ackermann RT, Finch EA, Brizendine E, Zhou H, Marrero DG. Translating the Diabetes Prevention Program into the Community. Am. J. Prev. Med. 2008;35:357–63.

22. Chinman M, Oberman RS, Hanusa BH, Cohen AN, Salyers MP, Twamley EW, et al. A Cluster Randomized Trial of Adding Peer Specialists to Intensive Case Management Teams in the Veterans Health Administration. J. Behav. Health Serv. Res. 2015;42:109–21.

23. Veterans Affairs Peer Specialists [Internet]. [cited 2013 Aug 28]. Available from: http://www.vacareers.va.gov/peer-to-peer/

24. Heisler M, Vijan S, Makki F, Piette JD. Diabetes Control With Reciprocal Peer Support Versus Nurse Care Management: A Randomized Trial. Ann. Intern. Med. 2010;153:507.

25. Heisler M. Different models to mobilize peer support to improve diabetes self-management and clinical outcomes: evidence, logistics, evaluation considerations and needs for future research. Fam. Pract. 2010;27:i23–i32.

26. McArthur D, Dumas A, Woodend K, Beach S, Stacey D. Factors influencing adherence to regular exercise in middle-aged women: a qualitative study to inform clinical practice. BMC Womens Health [Internet]. 2014 [cited 2017 May 12];14. Available from: http://bmcwomenshealth.biomedcentral.com/articles/10.1186/1472-6874-14-49

27. Lie MLS, Hayes L, Lewis-Barned NJ, May C, White M, Bell R. Preventing Type 2 diabetes after gestational diabetes: women’s experiences and implications for diabetes prevention interventions. Diabet. Med. 2013;30:986–93.

28. Brooks E, Dailey N, Bair B, Shore J. Rural Women Veterans Demographic Report: Defining VA Users’ Health and Health Care Access in Rural Areas: Rural Women Veterans. J. Rural Health. 2014;30:146–52.

29. Wantland DJ, Portillo CJ, Holzemer WL, Slaughter R, McGhee EM. The Effectiveness of Web-Based vs. Non-Web-Based Interventions: A Meta-Analysis of Behavioral Change Outcomes. J. Med. Internet Res. 2004;6:e40.

30. Kramer MK, McWilliams JR, Chen H-Y, Siminerio LM. A Community-Based Diabetes Prevention Program: Evaluation of the Group Lifestyle Balance Program Delivered by Diabetes Educators. Diabetes Educ. 2011;37:659–68.

31. Tang TS, Nwankwo R, Whiten Y, Oney C. Training Peers to Deliver a Church-Based Diabetes Prevention Program. Diabetes Educ. 2012;38:519–25.

32. Sepah SC, Jiang L, Peters AL. Translating the Diabetes Prevention Program into an Online Social Network: Validation against CDC Standards. Diabetes Educ. 2014;40:435–43.

33. CDC. National Diabetes Recognition Program (DPRP). [Internet]. Available from: www.cdc.gov/diabetes/prevention/recognition/about.htm.

34. Rogers EM. Diffusion of Innovations: Fifth Edition. New York: Free Press; 2003.

35. Hamilton. Qualitative methods in rapid turn-around health services research [Internet]. VA HSR&D Cyberseminar Spotlight on Women’s Health; 2013. Available from: http://www.hsrd.research.va.gov/for_researchers/cyber_seminars/archives/780-notes.pdf
