# Supplementary material for: Enhancing Mental and Physical Health of Women through Engagement and Retention (EMPOWER): a protocol for a program of research
Source: Implement Sci. 2017 Nov 7;12:127. doi: 10.1186/s13012-017-0658-9 (PMC5678767; doi:10.1186/s13012-017-0658-9)
Supplement: Supplementary file 2 — Individual project description: Facilitating Cardiovascular Risk Screening and Risk Reduction in Women Veterans (Bevanne Bean-Mayberry (PI) and Melissa Farmer (Co-PI)). (DOCX 61 kb) [file 13012_2017_658_MOESM2_ESM.docx]

**Project 2: Facilitating Cardiovascular Risk Screening and Risk Reduction in Women Veterans**

*Specific Aims*

Cardiovascular (CV) disease is the number one cause of death in American women, and all adult women are potentially at risk for CV disease [1,2]. While the American Heart Association (AHA) has documented that awareness of CV disease increased from 30% in 1997 to 54% in 2009, women still demonstrate limited understanding of the imminent risks of CV disease-associated morbidity and mortality. Recent updates summarizing the public health impact of CV disease in women identify the following topics as important issues: (a) greater need for prevention of incident cases of CV disease, (b) need for increased awareness of CV risk among women, and (c) need for larger representation of women in prevention trials [3,4]. The recently released CV guidelines for Cholesterol, CV Risk Assessment, Obesity and Lifestyle support directed lipid treatment and more personalized evaluations by providers, in addition to a strong push for educating patients about their risks and strongly recommending lifestyle changes (i.e., physical activity, diet, and behavioral counseling) [2,5–7]. There are clear gender differences in the control of CV risk factors such as lipids, blood pressure, and intermediate diabetes outcomes nationally and within the VA [8,9], with women Veterans often at higher CV risk than their male counterparts [3,10–15]. The combination of disparities and gender-specific CV risk factors suggest an urgent need for CV risk factor management in women Veterans.

The goals of this project are to implement and evaluate a CV risk reduction toolkit (CV toolkit) designed to increase identification of CV risk among Women Veterans, enhance patient/provider communication about their risk, and increase Women Veterans’ engagement and retention in relevant health services including referrals to key health programs (e.g., MOVE!, dieticians, health coaches, and CV specialists as needed). The multi-component toolkit is based on findings from our recently completed work for VA Women’s Health Services (WHS), in partnership with VA Greater Los Angeles (GLA) Women’s Health leadership, where we identified organizational barriers and facilitators to CV risk assessment and management in women Veterans. The CV toolkit incorporates the *Gateway to Healthy Living* program, a national program currently being implemented by VA National Center for Health Promotion and Disease Prevention (NCP) that focuses on motivating and supporting VA patients with CV risks to engage in existing VA services in order to reduce their risk and improve their health.

Guided by the Replicating Effective Programs (REP) framework, our specific aims are to:

1. Refine the elements of the CV toolkit, including patient education/activation tools, a CV risk assessment computerized template in CPRS, provider information/education and referral tools, and the *Gateway to Health Living* program specifically for Women Veterans;
2. Implement the CV Toolkit in four VA facilities with comprehensive women’s health clinics;
3. Evaluate toolkit implementation using a non-randomized stepped wedge design and conduct an implementation-focused evaluation to further refine the CV toolkit to facilitate future spread.

*Rationale*

CV risk reduction is hampered by provider- and patient-level barriers that are addressable with evidence-based implementation strategies. Provider barriers to working with patients on appropriate CV risk reduction include lack of time, lack of awareness of the latest CV disease prevention guidelines, difficulty interpreting the guidelines, difficulty accessing relevant electronic medical record (EMR) data at the point of care, low self-efficacy to counsel patients in behavioral change, habit or inertia, fragmentation of care, and perceptions of low patient interest or capacity to follow-through on recommendations [16–19]. These barriers may be decreased by provider education, training in the discussion and management of CV risk factors, centralization of relevant EMR information, patient activation support, and feedback to providers [16,18]. A meta-analysis of quality improvement (QI) efforts found that patient and provider education materials are effective at improving blood pressure control [20]. There is also evidence that clinical reminders are associated with better CV risk reduction practices in primary care populations [21–24]. In the VA, non-CV disease clinical reminders have similarly been effective at increasing screening (e.g., fall risk screening, HIV testing) [25,26]. While VA has multiple clinical reminders built into the Computerized Patient Record System (CPRS), this proposal merges existing data into a CV template and adds unique information for a more comprehensive screening and documentation process to facilitate the provider-patient discussion about each patient’s CV risks. Moreover, the information will be easily accessible in one CPRS location.

At the patient level, perception of personal CV risk remains limited but risk reduction interventions have been successful [27]. For example, gender- and non-gender-specific smoking cessation programs are effective at helping women abstain from smoking [28]. However, CV risk reduction challenges persist with weight management programs, showing varied results for effectiveness with changing caloric intake, affecting weight or BMI, or exercise capacity [11,29–33]. In women-specific programs, increased physical activity has led to reduced CV disease incidence and improved dietary changes [34–36]. Adherence and low-density lipoprotein (LDL) control can be improved by implementing low-cost or no-cost medications, increased levels of patient-focused education, improving the therapeutic alliance between patient and provider, and incorporating a pharmacist in primary care settings [37–41]. However, increasing evidence suggests that while some single outcome interventions are associated with small positive change, approaches that incorporate multiple strategies for patients may result in a more significant impact in populations needing primary or secondary CV prevention [42].

Health coaching holds promise for increasing patient engagement in appropriate services and lifestyle behaviors. Health coaching is defined as an on-going, patient-centered approach that involves collaboration with patients to help them develop internal motivation, experience self-discovery, increase self-efficacy, set goals, and achieve positive and lasting health behavior change [43]. In studies employing face-to-face in-person health coaching, patients improved diet, lost weight, increased physical activity, showed improvement in psychological markers such as self-efficacy, activation, stress and perceived barriers, and biological markers such as cholesterol and blood pressure, increased tobacco abstinence, and reduced cardiovascular risk factors overall [43–48]. Moreover, recent nursing literature reports that small program changes such as targeted emails and phone calls from nursing care managers can impact CV risk factors such as hypertension improvement among primary care patients [49]. For women patients specifically, a health coaching intervention with obese women increased attendance at an exercise group [47]. Another study found that women with type 2 diabetes who received a health coaching intervention had improved diet, physical activity, and weight [45]. Together, these findings point to the potential for structured, gender-specific coaching support to address CV risk among women Veterans. In this project we will capitalize on the NCP *Gateway to Healthy Living* program designed to serve as an entry point (or gateway) for interested Veterans to receive health information, motivational support, and collaborative goal setting to help with self-management for healthy living and use of relevant VA services [50].

Our team’s formative “pre-conditions” local work on CV risk reduction among women Veterans guides our proposed CV toolkit implementation research. In our FY2013 WHS-funded operations project, we 1) performed a targeted literature search on CV educational and risk assessment tools; 2) identified barriers and facilitators to CV risk assessment and risk reduction in women Veterans; and 3) identified key areas to target educational programs or tools to address needs in CV risk management. We conducted three focus groups with 21 Patient-Aligned Care Team (PACT) team members and semi-structured interviews with 19 patients at the two VA GLA primary care women’s clinics. Provider-identified barriers ranged from system difficulties in promoting prevention activities to communication challenges, limited time to dialog with patients, and limited patient knowledge. Provider-identified facilitators included strategies for effective patient engagement and motivation, tools and educational resources to aid CV risk discussions (especially in relation to co-morbidities), organized resources on CV health and available referrals, stronger integration of health coaches, and technology-based resources. Patient-identified barriers included poor motivation and competing demands, while facilitators included education about CV risks and complications in women, motivational and accountability support from others (including providers), and exercise programs that fit their lives and competing demands. Patients reported that various tools would be acceptable if their providers suggested using them, including paper tools, electronic tools, and in-person sources of support. Findings from this formative work indicate that evidence-based strategies need to be combined, tailored, and implemented at the local system level to facilitate provider-patient discussions, patient activation, and accountability to promote CV disease risk reduction for women Veterans.

*Procedures*

Implementation Strategy Overview. CV toolkit implementation will be guided by the Replicating Effective Programs (REP) framework [51] consistent with all EMPOWER QUERI projects, and will feature multilevel stakeholder engagement to ensure that each phase is informed by women Veterans, providers, administrators, and operations partners. The Tool for Evaluating Research Implementation Challenges (TECH) [37] will be used to guide assessment and documentation of implementation challenges during each REP phase.

Study Design, Site Selection and the CV Toolkit. We will use a non-randomized stepped wedge design to evaluate implementation and spread of the CV toolkit. We will use mixed methods to evaluate implementation. We will also use survey techniques and key stakeholder interviews with providers and patients to assess our primary implementation outcomes.

The CV toolkit will be implemented in four VA facilities with moderately large comprehensive WH clinics (Model 2 or 3 in VHA Handbook 1330.01). WH clinics will be eligible if they have primary care (PC) panels of women patients that total at least 2000+ women and if they have at least one PACT teamlet (comprised of primary care provider (PCP), registered nurse (RN), licensed vocational nurse (LVN), and health technician/MSA), availability of at least one other clinical staff member (e.g., dietitian, pharmacist or mental health provider, etc.), and 3 administrative staff (WH Medical Director, Women Veteran Program Manager, and Chief of Primary Care or PC Physician Leader). While multiple VA sites have women Veteran users in the thousands, sites for this study must have those patients linked to primary care providers in WH clinic settings.  At each site, we expect to have at least 4-8 PCPs per site who deliver care to women.  We will launch at GLA, where the PI (Bean-Mayberry) is the PBRN Site Lead. Three additional sites will be selected, aiming to involve one PBRN site per VISN.  Site selection will be done in collaboration with Dr. Susan Frayne, Director of the VA WH-PBRN, and member of our Strategic Advisory Group.

The CV Toolkit includes four components: (1) Patient education/activation tools, such as informational posters and fliers about women’s CV risk, including information about MyHealth*e*Vet, the website for Veterans to access their health records, schedule appointments, manage prescriptions, use secure messaging, and link to other resources. To activate patients to discuss CV risk with their providers, we developed a CV worksheet for patients to fill out at check-in to collect information about family history of CV disease, pregnancy/gestational history, and smoking status, and to help patients formulate/document any questions regarding their CV risks that they would like to discuss during their visits. The goal is to help make CV risk discussion a priority for women before they enter the exam room. (2) A CV risk assessment computerized template systematically captures CV disease risk factor history and data from the medical record, and provides open fields for the provider to enter any relevant information from the patient worksheet and the clinic visit (e.g., goals and decisions). The template also includes embedded links to locate CV guideline documents. Based on feedback from clinical teams at two GLA sites, the template includes data from the current visit as well as the last three entries in the medical record (automatically generated by the template) such as weight, blood pressure, and cholesterol lab results. This enables the provider to look at trends in risk factors without having to search the chart. The information is stored as a standard outpatient note in the patient’s medical record (in CPRS) and is readily accessible (and searchable) at future visits so that CV risk status will be an ongoing discussion. Referral options are also included on the template, including referral to the *Gateway to Healthy Living* program, so the template will be a shared template available for other providers to access (e.g. health coach, dietician, specialist, etc.) to review when the patient comes for the referral [52]. (3) Provider information and education programs as well as referral tools to internal services (e.g., women’s MOVE! program, smoking cessation clinics, dieticians, health coaches, pharmacists and CV or mental health specialists as needed). (4) The *Gateway to Healthy Living* program tailored to Women Veterans is a facilitated group meeting that occurs in a structured format with personalized goal setting and identification of key services available at the local site to assist with lifestyle changes necessary for patient-targeted CV risk reduction. In addition to the structured group, it includes at least two follow up phone calls from the facilitator (typically a health coach or health promotion disease prevention specialist) for support, reinforcement of lifestyle changes, brainstorming of barriers, and steps toward goal realization.

*REP implementation phases*

Pre-conditions (Aim 1): In our prior work (funded by WHS), the need for the intervention was established and review of effective interventions completed. We will have the completed the refinement, programming, functionality testing and final loading of the CPRS-based template in the CV toolkit package before project launch. We are also culling VA and AHA information on CV disease in women and testing online VA media for patient assessments in myHealtheVet. We have also been working with our partners at NCP and identified the *Gateway to Healthy Living* program for in-person, patient-directed goal setting. We will work closely with NCP to evaluate the results from the current *Gateway to Healthy Living* pilot project (concluding FY15) to make program adjustments and further tailor the program for women Veterans.

Furthermore, the pre-conditions phase has been completed locally at GLA, which will serve as an initial implementation site. When additional Women’s Health Practice Based Research Network (PBRN) sites are selected, we will revisit aspects of the pre-conditions phase to ensure that the toolkit, developed locally at GLA, is appropriate at the other sites. During site visits at the additional sites, the toolkit will be discussed with relevant key stakeholders (WH leaders, providers, women Veteran patient representatives), core elements will be explained, and, using the TECH, the local team will discuss options for adapting delivery and anticipated barriers to implementation. For example, we will need to explore care options available at each site (e.g. health coaches, smoking cessation, MOVE!, etc.) and educational needs of the various WH teams at each site. Also during this phase, interviews and surveys will be conducted with all consenting key stakeholders, with the exception of patients who will be interviewed and surveyed in the implementation phase (see below).

Pre-implementation (Aim 1 cont.): At each site, a local CV toolkit champion will be identified by the Site Lead when s/he signs onto the project. The toolkit package will be further developed and locally tailored, with attention to training and technical assistance needed for each site. Once prepared, the toolkit will be piloted locally to ensure that it works as intended with local systems and processes. Using the TECH, any challenges with pilot implementation will be discussed and addressed by the team. When the team determines that challenges have been sufficiently addressed, orientation meetings will be held with the broader clinic, where the toolkit will be distributed and discussed.

Implementation (Aim 2): The implementation phase will begin at two PBRN sites in the latter half of Year 2 of the EMPOWER QUERI and will last a total of 15 months to ensure adequate time for implementation evaluation and reach. The toolkit will spread to two additional PBRN sites in Year 3. For CV toolkit training, we will lead a series of provider education activities including facilitated discussions to educate the providers and office staff members on the importance of CV risk discussions for women within primary care as well as on how to use the toolkit in practice. At GLA, this training will be done in person, but the training programs will be tailored for virtual delivery (e.g. Lync meeting/cyberseminars) to the other sites. In addition, our partners at NCP will travel to all sites to train the *Gateway to Healthy Living* facilitators. During implementation, we will assess any additional need for provider training and monitor the use of the template in the clinic by generating reports on template use. This information will be collected by the research staff, the data will be summarized (frequencies graphed over time), and then quarterly CV template reports will be presented to the WH clinical team. We will also take field notes during the trainings to document any issues that arise with the users (clinical team) and the CV toolkit within the context of each distinct clinic setting. These notes will be analyzed using ATLAS.ti, and will be examined in conjunction with other evaluation data as described below. During regular implementation meetings, TECH will be used to assess and address implementation challenges.

The CV toolkit is an innovative care model that is proactive, patient-centered and personalized to each woman that comes to an appointment. A patient begins using the CV toolkit at check-in for her appointment when she is given the CV worksheet to fill out while waiting to see her provider. Patient education materials specifically on women and CV risk will be in the waiting room and exam rooms. The CV worksheet will be used to initiate the provider-patient discussion of CV risk, and her provider will use the information to fill out the CV template in CPRS during the appointment. The patient and provider will collaboratively determine the next steps (i.e. *Gateway to Healthy Living*, other referral, or waiting) and the provider will document the action plan in the template. All participants will be offered the *Gateway to Healthy Living* program by providers as engagement and support for the patient action plan. After the appointment, each woman will be asked if she is willing to participate in the project for monitoring CV risks and choices and if she consents, she will complete a patient survey with the RA. Those women who are referred to that *Gateway to Healthy Living* program will be contacted by the program facilitator immediately to arrange the group meeting. The women will be considered engaged in the *Gateway to Healthy Living* program when she attends the facilitated group meeting. Information on goal setting, behavior change intentions and participation in other services will be collected by the facilitator at the group meeting and at the follow-up phone calls. Follow-up surveys will be administered to all women who completed the baseline survey six months after the baseline survey.

Maintenance and Evolution: The last phase in this framework allows us to take the feedback and make modifications to our implementation process to enhance adoption of the toolkit, fidelity to the implementation process, and dissemination at future sites. Sharing these details with stakeholders using TECH informs the current implementation process and sustainability for the future. The four REP phases allow us to account for each step of the guided implementation strategy. Finally, the interchange between these phases and the implementation process components provide a granular synergy for understanding whether the implementation of this CV toolkit requires further adaptation and customization prior to broader VA dissemination. During this phase, the research team will collaborate with the local implementation teams to develop points for the CV Toolkit Implementation Playbook.

*Implementation Evaluation*

Mixed Methods Data Collection. Mixed methods will be used to evaluate implementation of the CV toolkit (Aim 3). During the pre-conditions phase, key informants will be asked complete a brief survey of organizational climate for implementation. Semi-structured interviews will be conducted with key stakeholders (n~15 per site) at the sites, including WH primary care providers, nurses, clinical staff and health coaches, to evaluate knowledge, attitudes, beliefs, and perceived acceptability and feasibility of the toolkit (approximately five months into the pre-conditions phase); perceptions of interim implementation processes and outcomes (approx. six months into the implementation phase) and summative perceptions of toolkit implementation and potential for spread (last two months of the maintenance/evolution phase). Key stakeholders who will be directly involved in using the toolkit will also be asked to complete the Perceived Characteristics of Intervention Scale. Also, the CV template reports (discussed above) will enable the clinical team along with the research team to use TECH to troubleshoot any problems with uptake, acceptability and usability of the template and make appropriate changes.

We will collect baseline patient information by asking each patient (n=130) to participate in our project after her appointment. Those who agree will complete informed consent and then be asked to complete a survey after the PCP appointment or at another convenient time. Those who complete the baseline survey will receive $10. They will be asked for contact information in order to reach them for a 6-month follow-up survey, for which they will receive another $10. A subset of patients (approximately 45 per site, or every third consented patient) will be asked to participate in a brief semi-structured interview at baseline ($10) and six months post-baseline ($10). For those patients who participate in the *Gateway to Healthy Living* program, additional information on goal-setting and program participation will be collected after completing the program.

Measures. Primary implementation outcomes for this project will be consistent with the implementation outcomes for all projects, i.e., adoption, acceptability, feasibility, and penetration/reach. Additional project-specific measures will also be included. For providers, interview questions will be designed to understand how the CV toolkit impacts a number of domains including: comfort and ease of communication discussing personal CV risk with women Veterans, perceived patient awareness of CV risk and activation, tracking and management of personal CV risk factors (such as BMI and blood pressure) over time, relative priority and compatibility of the CV toolkit within the general workflow, competing demands, and ease of referral to the *Gateway to Health Living* program, as well as other barriers and facilitators associated with toolkit implementation. Measures of provider template use (percent of patients the provider used the CPRS template) and number of CV-related referrals (total count of CV related referrals, *Gateway to Healthy Living-*specific referrals and other services) will be captured from the Corporate Data Warehouse (CDW). For each referral, we will create a dichotomous variable (1=yes referral made). We will use these dichotomous variables to create a count measure of total referrals.

For patients, project-specific interview questions will focus on perceptions of CV risk and barriers and facilitators to behavior change. Readiness to quit measures will be included for smokers. Patient data will also be extracted from CDW for all patients seen by the provider in the twelve months following the provider’s engagement in the toolkit. Measures include blood pressure, weight, height cholesterol panel (including LDL & HDL), hemoglobin A1c, as well as any incidence of cardiovascular events (e.g. stroke, MI) or cardiac procedures (cardiac catheterization, stress test, cardiology consultations). Patient demographics and co-morbidity diagnoses from CDW will serve as controls in the analyses. For *Gateway to Healthy Living* patients, we will also measure attendance at the facilitated group (1=yes) and follow-up contact completion (number of completed contacts), identified health behavior goal(s), details of action plan/ health behavior change SMART goal, change in confidence ratings for behavior change, and satisfaction with program.

*Analysis*

Qualitative Analysis*:* In addition to the general EMPOWER QUERI approach to qualitative analysis, for providers, we will compare and contrast data from frequent users of the template with infrequent users, and track changes in perceptions about the utility and impact of the toolkit on clinical practice from pre-conditions to maintenance. We will also assess provider attitudes toward ease of referral to the *Gateway to Healthy Living* program, and provider perceptions of changes in patient awareness and activation. For patients, we will compare and contrast data from those who used *Gateway to Healthy Living* with those who did not, and those who used other services. In order to evaluate the patient impact of *Gateway to Healthy Living*, we will assess changes in patient perceptions of CV risk, self-efficacy, and activation.

Quantitative Analysis: We will use descriptive and inferential statistics to understand variations in the measures described above. For patients, we will conduct bivariate analysis to examine variations in outcomes (CV risk factor control, cardiac event or procedure, etc.) by those who participated in the *Gateway to Healthy Living* program, versus those who received a referral to a different program/service, and those who received no referral. For providers, we will examine differences in their patient panel (e.g. those with CV risk factors under control at follow-up) by their engagement in the CV toolkit program. To evaluate the CV toolkit, we will use the non-randomized stepped wedge design, a generalized mixed model coupled with an allowance for nonrandom effects, to evaluate the implementation across the providers at four sites. The intervention will be defined as “turning on” when a provider first engages in the CV toolkit (first uses the CV template). We will model the effect of the implementation of the CV toolkit intervention on referral rates, while controlling for organizational level, provider level and patient level covariates.

*Impact*

Refining, implementing, and evaluating the innovative CV toolkit to initiate and facilitate appropriate screening, discussion, and documentation of CV risk is a first step for improving patient-provider conversations about CV risk factors and actionable steps to manage risk factors. The *Gateway to Healthy Living* Program builds on this process by incorporating a structured facilitated group session for the patient to set specific lifestyle goals for CV risk reduction, map out intended steps and learn about health care services available at their local site which can support and reinforce their goal setting through coordinated short term telephone follow up from the facilitator. Overall, the CV toolkit is an innovative care model that is designed to achieve the EMPOWER QUERI goal of enriching organizational capacity in order to improved engagement and retention of women Veterans in patient-centered, proactive, personalized care—in this project, care that addresses CV risk. The toolkit components encourage Veterans to become more active participants in their health care by improving communication and interactions between health care providers and Veterans and giving women choices as to how to achieve their health goals. Nationally, CV risks are pervasive with persistent differences by gender; eliminating these disparities and reducing overall risks is a priority for all of our operations partners.

*Limitations*

The CV toolkit will be implemented at four comprehensive WH clinics where the majority of the female Veterans at these sites are likely to receive primary care [53] in separate WH clinics. We will not be able to generalize to the sites that have implemented care for women Veterans with one of the other VHA Handbook 1330.0 sanctioned WH care delivery models (e.g. designated WH PC provider within general PC or separate from PC). The results from the current proposal will be foundational for future interventions to address similar engagement innovations or ones that include other facilitators using technology (e.g., smart phone applications, email notifications) and barriers for CV risk reduction related to medication initiation, titration and patient adherence. For now, this study will evaluate patient-level intermediate outcomes that are secondary to our main aims (i.e., changes in blood pressure, weight, body mass index, cholesterol level, etc.), which will inform our team about effectiveness while using engagement innovations specifically targeted to women Veterans. Such studies will provide insight for tailoring women’s CV care within VA and nationally.

This CV toolkit aligns directly with both VA WHS’s mission to provide comprehensive health care for women based on provider competencies and patient preferences, and NCP’s disease prevention goals for all Veterans. Moreover, per the EMPOWER QUERI conceptual framework, the toolkit—a personalized, proactive-patient driven care model (consistent with the VA Blueprint for Excellence)—represents an innovation in women Veterans’ health care that will enrich organizational capacity to engage and retain women Veterans in appropriate care, thereby reducing their risk. This particular project will leverage information technology (CPRS templates, NCP resources and VA online tools for Veteran education (e.g., myHealtheVet)) and models of healthcare delivery to optimize individual and population-based health outcomes.

**References**

1. Mosca L, Benjamin EJ, Berra K, Bezanson JL, Dolor RJ, Lloyd-Jones DM, et al. Effectiveness-Based Guidelines for the Prevention of Cardiovascular Disease in Women--2011 Update: A Guideline From the American Heart Association. Circulation. 2011;123:1243–62.

2. Goff DC, Lloyd-Jones DM, Bennett G, Coady S, D’Agostino RB, Gibbons R, et al. 2013 ACC/AHA Guideline on the Assessment of Cardiovascular Risk: A Report of the American College of Cardiology/American Heart Association Task Force on Practice Guidelines. Circulation. 2014;129:S49–S73.

3. Jarvie JL, Foody JM. Recognizing and Improving Health Care Disparities in the Prevention of Cardiovascular Disease in Women. Curr. Cardiol. Rep. 2010;12:488–96.

4. Melloni C, Berger JS, Wang TY, Gunes F, Stebbins A, Pieper KS, et al. Representation of Women in Randomized Clinical Trials of Cardiovascular Disease Prevention. Circ. Cardiovasc. Qual. Outcomes. 2010;3:135–42.

5. Stone NJ, Robinson JG, Lichtenstein AH, Bairey Merz CN, Blum CB, Eckel RH, et al. 2013 ACC/AHA Guideline on the Treatment of Blood Cholesterol to Reduce Atherosclerotic Cardiovascular Risk in Adults: A Report of the American College of Cardiology/American Heart Association Task Force on Practice Guidelines. Circulation. 2014;129:S1–S45.

6. Jensen MD, Ryan DH, Apovian CM, Ard JD, Comuzzie AG, Donato KA, et al. 2013 AHA/ACC/TOS Guideline for the Management of Overweight and Obesity in Adults: A Report of the American College of Cardiology/American Heart Association Task Force on Practice Guidelines and The Obesity Society. Circulation. 2014;129:S102–S138.

7. Eckel RH, Jakicic JM, Ard JD, de Jesus JM, Miller NH, Hubbard VS, et al. 2013 AHA/ACC Guideline on Lifestyle Management to Reduce Cardiovascular Risk: A Report of the American College of Cardiology/American Heart Association Task Force on Practice Guidelines. Circulation. 2014;129:S76–S99.

8. Deedwania P, Lardizabal. Benefits of statin therapy and compliance in high risk cardiovascular patients. Vasc. Health Risk Manag. 2010;843.

9. Chou AF, Wong L, Weisman CS, Chan S, Bierman AS, Correa-de-Araujo R, et al. Gender Disparities in Cardiovascular Disease Care Among Commercial and Medicare Managed Care Plans. Womens Health Issues. 2007;17:139–49.

10. Bean-Mayberry B, Yano EM, Mor MK, Bayliss NK, Xu X, Fine MJ. Does Sex Influence Immunization Status for Influenza and Pneumonia in Older Veterans?: GENDER AND IMMUNIZATIONS IN OLDER VETERANS. J. Am. Geriatr. Soc. 2009;57:1427–32.

11. Vimalananda VG, Miller DR, Hofer TP, Holleman RG, Klamerus ML, Kerr EA. Accounting for Clinical Action Reduces Estimates of Gender Disparities in Lipid Management for Diabetic Veterans. J. Gen. Intern. Med. 2013;28:529–35.

12. Tseng C-L, Sambamoorthi U, Rajan M, Tiwari A, Frayne S, Findley P, et al. Are there gender differences in diabetes care among elderly medicare enrolled veterans? J. Gen. Intern. Med. 2006;21:S47–S53.

13. Jha AK, Perlin JB, Steinman MA, Peabody JW, Ayanian JZ. Brief report: Quality of ambulatory care for women and men in the veterans affairs health care system. J. Gen. Intern. Med. 2005;20:762–5.

14. LaMarca BD, Gilbert J, Granger JP. Recent Progress Toward the Understanding of the Pathophysiology of Hypertension During Preeclampsia. Hypertension. 2008;51:982–8.

15. Farmer MM, Rose DE, Riopelle D, Lanto AB, Yano EM. Gender Differences in Smoking and Smoking Cessation Treatment: An Examination of the Organizational Features Related to Care. Womens Health Issues. 2011;21:S182–S189.

16. Cabana MD, Kim C. Physician adherence to preventive cardiology guidelines for women. Womens Health Issues. 2003;13:142–9.

17. Crosson JC, Heisler M, Subramanian U, Swain B, Davis GJ, Lasser N, et al. Physicians’ Perceptions of Barriers to Cardiovascular Disease Risk Factor Control among Patients with Diabetes: Results from the Translating Research into Action for Diabetes (TRIAD) Study. J. Am. Board Fam. Med. 2010;23:171–8.

18. Kedward J, Dakin L. A qualitative study of barriers to the use of statins and the implementation of coronary heart disease prevention in primary care. Br. J. Gen. Pract. 53:684–9.

19. Tsui J, Dodson K, Jacobson T. Cardiovascular disease prevention counseling in residency: resident and attending physician attitudes and practices. J. Natl. Med. Assoc. 2004;96:1080–3.

20. Walsh JME, Sundaram V, McDonald K, Owens DK, Goldstein MK. Implementing effective hypertension quality improvement strategies: barriers and potential solutions. J. Clin. Hypertens. Greenwich Conn. 2008;10:311–6.

21. Kenealy T, Arroll B, Petrie KJ. Patients and computers as reminders to screen for diabetes in family practice: Randomized-controlled trial. J. Gen. Intern. Med. 2005;20:916–21.

22. McMenamin SB, Bellows NM, Halpin HA, Rittenhouse DR, Casalino LP, Shortell SM. Adoption of policies to treat tobacco dependence in U.S. medical groups. Am. J. Prev. Med. 2010;39:449–56.

23. Shea S, DuMouchel W, Bahamonde L. A meta-analysis of 16 randomized controlled trials to evaluate computer-based clinical reminder systems for preventive care in the ambulatory setting. J. Am. Med. Inform. Assoc. JAMIA. 1996;3:399–409.

24. Shojania KG, Jennings A, Mayhew A, Ramsay C, Eccles M, Grimshaw J. Effect of point-of-care computer reminders on physician behaviour: a systematic review. Can. Med. Assoc. J. 2010;182:E216–E225.

25. Spears GV, Roth CP, Miake-Lye IM, Saliba D, Shekelle PG, Ganz DA. Redesign of an Electronic Clinical Reminder to Prevent Falls in Older Adults: Med. Care. 2013;51:S37–S43.

26. Czarnogorski M, Halloran, CNS J, Pedati C, Dursa EK, Durfee J, Martinello R, et al. Expanded HIV Testing in the US Department of Veterans Affairs, 2009–2011. Am. J. Public Health. 2013;103:e40–e45.

27. Mosca L, Mochari-Greenberger H, Dolor RJ, Newby LK, Robb KJ. Twelve-Year Follow-Up of American Women’s Awareness of Cardiovascular Disease Risk and Barriers to Heart Health. Circ. Cardiovasc. Qual. Outcomes. 2010;3:120–7.

28. Turnbull F, Woodward M, Neal B, Barzi F, Ninomiya T, Chalmers J, et al. Do men and women respond differently to blood pressure-lowering treatment? Results of prospectively designed overviews of randomized trials. Eur. Heart J. 2008;29:2669–80.

29. Davis M, Duvernoy C. How to stay heart healthy in 2011: considerations for the primary prevention of cardiovascular disease in women. Womens Health Lond Engl. 2011;7:433–51.

30. Church TS, Earnest CP, Skinner JS, Blair SN. Effects of Different Doses of Physical Activity on Cardiorespiratory Fitness Among Sedentary, Overweight or Obese Postmenopausal Women With Elevated Blood Pressure: A Randomized Controlled Trial. JAMA. 2007;297:2081.

31. Anderson D, Mizzari K, Kain V, Webster J. The Effects of a Multimodal Intervention Trial to Promote Lifestyle Factors Associated With the Prevention of Cardiovascular Disease in Menopausal and Postmenopausal Australian Women. Health Care Women Int. 2006;27:238–53.

32. Parra-Medina D, Wilcox S, Salinas J, Addy C, Fore E, Poston M, et al. Results of the Heart Healthy and Ethnically Relevant Lifestyle Trial: A Cardiovascular Risk Reduction Intervention for African American Women Attending Community Health Centers. Am. J. Public Health. 2011;101:1914–21.

33. LeBlanc E, O’Connor E, Whitlock EP, Patnode C, Kapka T. Screening for and Management of Obesity and Overweight in Adults [Internet]. Rockville (MD): Agency for Healthcare Research and Quality (US); 2011 [cited 2017 May 11]. Available from: http://www.ncbi.nlm.nih.gov/books/NBK65294/

34. Leykum LK, Pugh JA, Lanham HJ, Harmon J, McDaniel RR. Implementation research design: integrating participatory action research into randomized controlled trials. Implement. Sci. [Internet]. 2009 [cited 2017 May 11];4. Available from: http://implementationscience.biomedcentral.com/articles/10.1186/1748-5908-4-69

35. Jordon M, Lanham HJ, Anderson RA, McDaniel Jr RR. Implications of complex adaptive systems theory for interpreting research about health care organizations. J. Eval. Clin. Pract. 2010;16:228–31.

36. Yano EM, Haskell S, Hayes P. Delivery of Gender-Sensitive Comprehensive Primary Care to Women Veterans: Implications for VA Patient Aligned Care Teams. J. Gen. Intern. Med. 2014;29:703–7.

37. Simpson KM, Porter K, McConnell ES, Colón-Emeric C, Daily KA, Stalzer A, et al. Tool for evaluating research implementation challenges: A sense-making protocol for addressing implementation challenges in complex research settings. Implement. Sci. [Internet]. 2013 [cited 2017 May 11];8. Available from: http://implementationscience.biomedcentral.com/articles/10.1186/1748-5908-8-2

38. Ehrhart MG, Aarons GA, Farahnak LR. Assessing the organizational context for EBP implementation: the development and validity testing of the Implementation Climate Scale (ICS). Implement. Sci. [Internet]. 2014 [cited 2017 May 11];9. Available from: http://implementationscience.biomedcentral.com/articles/10.1186/s13012-014-0157-1

39. Aarons GA, Ehrhart MG, Farahnak LR. The implementation leadership scale (ILS): development of a brief measure of unit level implementation leadership. Implement. Sci. [Internet]. 2014 [cited 2017 May 11];9. Available from: http://implementationscience.biomedcentral.com/articles/10.1186/1748-5908-9-45

40. Ehrhart MG, Aarons GA, Farahnak LR. Going above and beyond for implementation: the development and validity testing of the Implementation Citizenship Behavior Scale (ICBS). Implement. Sci. [Internet]. 2015 [cited 2017 May 11];10. Available from: http://implementationscience.biomedcentral.com/articles/10.1186/s13012-015-0255-8

41. Oostendorp LJ, Durand M-A, Lloyd A, Elwyn G. Measuring organisational readiness for patient engagement (MORE): an international online Delphi consensus study. BMC Health Serv. Res. [Internet]. 2015 [cited 2017 May 11];15. Available from: http://bmchealthservres.biomedcentral.com/articles/10.1186/s12913-015-0717-3

42. Goldstein KM, Melnyk SD, Zullig LL, Stechuchak KM, Oddone E, Bastian LA, et al. Heart Matters: Gender and Racial Differences Cardiovascular Disease Risk Factor Control Among Veterans. Womens Health Issues. 2014;24:477–83.

43. Wolever RQ, Dreusicke M, Fikkan J, Hawkins TV, Yeung S, Wakefield J, et al. Integrative Health Coaching for Patients With Type 2 Diabetes. Diabetes Educ. 2010;36:629–39.

44. Eakin E, Reeves M, Lawler S, Graves N, Oldenburg B, Del Mar C, et al. Telephone Counseling for Physical Activity and Diet in Primary Care Patients. Am. J. Prev. Med. 2009;36:142–9.

45. Whittemore R, Melkus GD, Sullivan A, Grey M. A Nurse-Coaching Intervention for Women With Type 2 Diabetes. Diabetes Educ. 2004;30:795–804.

46. Smith BN, Vaughn RA, Vogt D, King DW, King LA, Shipherd JC. Main and interactive effects of social support in predicting mental health symptoms in men and women following military stressor exposure. Anxiety Stress Coping. 2013;26:52–69.

47. Annesi JJ, Unruh JL, Marti NC, Gorjala S, Tennant G. Effects of The Coach Approach Intervention on Adherence to Exercise in Obese Women: Assessing Mediation of Social Cognitive Theory Factors. Res. Q. Exerc. Sport. 2011;82:99–108.

48. Edelman D, Oddone EZ, Liebowitz RS, Yancy WS, Olsen MK, Jeffreys AS, et al. A multidimensional integrative medicine intervention to improve cardiovascular risk. J. Gen. Intern. Med. 2006;21:728–34.

49. Bosworth H. A nurse-led email reminder programme on healthy lifestyle can improve cardiovascular risk factors in hypertensive adults. Evid. Based Nurs. 2015;18:56–56.

50. Gateway to Healthy Living Framework, VA NCP.

51. Kilbourne AM, Neumann MS, Pincus HA, Bauer MS, Stall R. Implementing evidence-based interventions in health care: application of the replicating effective programs framework. Implement. Sci. [Internet]. 2007 [cited 2017 May 11];2. Available from: http://implementationscience.biomedcentral.com/articles/10.1186/1748-5908-2-42

52. Daumit GL, Dickerson FB, Wang N-Y, Dalcin A, Jerome GJ, Anderson CAM, et al. A Behavioral Weight-Loss Intervention in Persons with Serious Mental Illness. N. Engl. J. Med. 2013;368:1594–602.

53. Hassija CM, Jakupcak M, Maguen S, Shipherd JC. The Influence of combat and interpersonal trauma on PTSD, depression, and alcohol misuse in U.S. Gulf War and OEF/OIF women veterans. J. Trauma. Stress. 2012;25:216–9.
