# Supplementary material for: Enhancing Mental and Physical Health of Women through Engagement and Retention (EMPOWER): a protocol for a program of research
Source: Implement Sci. 2017 Nov 7;12:127. doi: 10.1186/s13012-017-0658-9 (PMC5678767; doi:10.1186/s13012-017-0658-9)
Supplement: Supplementary file 3 — Implementation of Tailored Collaborative Care for Women Veterans (Alison Hamilton (PI) and Ariel Lang (Co-PI)). (DOCX 50 kb) [file 13012_2017_658_MOESM3_ESM.docx]

**Project 3: Implementation of Tailored Collaborative Care for Women Veterans (CCWV)**

*Specific Aims*

Almost half (48%) of women Veterans have at least one mental health (MH) condition [1], with depression as the most common MH condition among women Veteran VA users (24% in FY14), followed by PTSD (14%) and anxiety (8%). Depression and anxiety are substantially more prevalent among women Veterans than men [2]. Women Veterans are also more likely than men to have comorbid MH conditions, and they are more likely to have experienced military sexual trauma (MST) [3,4] and intimate partner violence (IPV) [5]. Like their male counterparts, women Veterans tend to have complex mental health needs. However, in contrast with men, a significant proportion of women’s symptoms, traumas, and treatment contraindications may be related to reproductive phases and experiences, gendered trauma histories, and gender role responsibilities.

Women Veterans’ distinctive MH needs challenge the organization to appropriately connect women to MH providers who understand their unique vulnerabilities and strengths, and to support their navigation of often fragmented MH care options offered across VA primary care (PC) and MH settings. Depending on the VA site, women may have access to integrated MH care in general PC clinics, in a separate portion of a general PC clinic set aside for women, or in stand-alone women-only clinics, with variable access to women-only care options and same-gender providers when preferred. MH clinics may have no special arrangements for women, or may have some providers to whom women are preferentially assigned. At a few sites, women-only MH clinics may be available, or if the site has a comprehensive women’s PC clinic, full-service specialty MH care may be co-located [6]. Such highly variable models of MH care delivery in PC and MH further challenge the system to engage women in services in a timely manner, and to support their retention in care.

Collaborative care models have a strong evidence base for enhancing patient engagement and retention in PC-based MH care for depression [7–9], and the VA has extended application of collaborative care to many other mild to moderate conditions, including anxiety disorders and alcohol misuse. The VA’s approach to integrated MH involves collaborative care with two components: co-located MH professionals who are integral components of the PC team, and MH care management [10]. While co-location has been achieved at many sites, implementation of care management has been limited, especially in women’s clinics. The VA endorses three collaborative care models that emphasize different elements of care management: Behavioral Health Lab [11], White River Junction [12], and Translating Initiatives in Depression into Effective Solution (TIDES) [13]. A recent report on Integrating Mental Health into VA’s Patient-Aligned Care Teams (PACT) [14] suggests that further work should be directed at understanding the advantages and disadvantages of each approach, and suggests moving away from branded approaches, toward blended models with local tailoring.

VA policy specifies that MH services must be provided in a manner that recognizes that gender-specific issues can be important components of care (1660.01). With attention to gender-specific concerns, collaborative care is a promising model to improve PC-MH integration for women Veterans. We propose implementation of tailored “Collaborative Care for Women Veterans” (CCWV) that encourages local blending of elements from VA-approved models with a PC-friendly evidence-based, computer-assisted cognitive behavioral treatment (CBT) platform for anxiety, PTSD and depression [15].

Our specific aims are to:

1. In four PBRN sites, blend and tailor existing primary care-based collaborative care models with an evidence-based CBT platform to provide evidence-based, patient-centered collaborative care for women Veterans (CCWV) with anxiety, PTSD and/or depression;
2. Implement the tailored CCWV in the four PBRN sites; and
3. Evaluate CCWV implementation using a modified stepped wedge design and conduct an implementation-focused evaluation to further refine CCWV to facilitate future spread.

Achieving these aims will help achieve VA comprehensive PC policies for women Veterans and VA MH Strategic Analytics for Improvement and Learning (SAIL) performance goals of population coverage, continuity of care, and experience of care.

Our secondary aims focus on factors that empower Women Veterans to engage in and benefit from collaborative care. Specifically, we will evaluate a) effects of patient activation and engagement with care on retention in and experiences of CCWV, and b) changes in MH symptoms, patient activation, health-related quality of life, and care experiences as a function of participation in CCWV. We hypothesize that a) more highly activated women and women with greater increases in activation will demonstrate better retention in care and report better care experiences, and b) symptoms, patient activation and quality of life will improve as a function of CCWV participation.

*Rationale*

The rationale for this project is based on five fundamental premises: 1) PC and MH care for women Veterans are complex and diverse (as described above); 2) treatment of anxiety, PTSD and/or depression in PC should be part of the continuum of care; 3) improvements are needed in PC-MHI for women in these complex care structures and settings; 4) gender-sensitive collaborative care would benefit the VA, providers, and women Veterans themselves; and 5) blended models of existing collaborative care models or components, with some enhancements, are needed in order to fit with local WH structures, needs, and resources.

Anxiety, PTSD and depression among women Veterans can and should be treated in the PC context. Since 2007, Primary Care-Mental Health Integration (PC-MHI) in VHA has evolved into a requirement at all VHA medical centers and large community-based outpatient clinics [16]. VA WH policy now requires that basic mental health services be provided in the same physical location as women’s primary care, whether in a gender-neutral primary care clinic, in shared separate space in the gender-neutral clinic, or in a separate WH center. In addition, WH clinics must have co-located MH and social work services. Assessment and psychosocial treatment need to be available in women’s PC for MH disorders, including depression and problem drinking, within the parameters of PC-MHI.

Improvements are needed in PC-MHI for women in VA’s complex WH care structures and settings. Women’s Assessment Tool for Comprehensive Health (WATCH) data provided to us by our WHS primary partners indicates that roughly 63% of VA healthcare settings (including medical centers and community-based outpatient clinics) have availability of PC-MHI such that a woman can “see a PC-MHI provider when clinically indicated…whenever this clinic is open to see patients on a timely, same-day basis.” Unpublished interview data from several of our team’s qualitative key informant studies suggest that MH services available in women’s PC (or in an adjacent women’s MH clinic) tend to be longer-term specialty MH services (as opposed to PC-MHI assessments and brief therapies) as well as women-only treatment groups, particularly for MH problems associated with MST experience. Informants have tended to report good communication between PC and MH providers, with “warm hand-offs” and curb-siding easily occurring in the clinic, but without a care management component to assess and triage the more and less severe cases to appropriate services.

True integration of MH supports for PC-based management of mild to moderate MH conditions may be overshadowed by co-located specialty MH services for more serious conditions. On the other hand, when women receive their PC in mixed-gender general PC clinics, women’s proportional minority creates challenges to provision of integrated services that can provide choice of women-only and same-gender provider services when desired, and may limit access to integrated MH providers experienced in working with the unique trauma experiences of women in the military. Varying care arrangements and varying needs for gender-specific choices make the “glue” of a care management process very important to help women Veterans access appropriate services, while also making care management more difficult to implement.

Furthermore, work from the VA Women’s PBRN [17] indicates that depression and PTSD are associated with women’s strong preferences for designated women’s mental health services, as well as treatment within the PC setting. According to a recent national survey of women Veterans, access to MH care for these conditions is good, but nearly half of respondents reported that VA MH services do not fully meet their needs [17]. Our WH, MH, and PC partners have urged us to use this QUERI implementation research opportunity to improve PC-MHI for women Veterans with depression, anxiety and/or PTSD.

Collaborative care designed for women Veterans with depression, anxiety and/or PTSD would benefit the VA, providers, and women Veterans themselves. Numerous similar models of collaborative care have been tested, primarily for care of depression in primary care settings, both outside the VA [18,19], and in military [20] and VA [11,12,21] populations. A 2006 meta-analysis found that collaborative care is more effective than standard care in improving depression outcomes in the short and longer terms, and concluded that future research needs to address the implementation of collaborative care [7].

The overall higher MH burden among women Veterans, and greater complexity of presentations, adds to the challenges of tailoring care management to meet women’s needs. Models based on treating one MH condition, such as depression, tend to exclude patients with more complex issues. Our recent work has found that over one-quarter (27%) of women Veteran VA users have two or more MH conditions. Though depression is the most common MH diagnosis among women Veterans, depression very often presents together with other disorders; in fact, depression-plus-anxiety and depression-plus-PTSD are tied for the two most prevalent co-occurring MH conditions among women Veterans [1]. Therefore, collaborative care management models that incorporate PC-friendly transdiagnostic brief treatment options for co-morbid presentations may be particularly suited to women Veteran populations. In addition, for those women whose MH needs exceed what can be offered in PC, strategies to support engagement in specialty care (especially when women-only settings are not available), as well as strong stepped care and referral management components will be important.

Because of the need for transdiagnostic and flexible models to meet women’s MH needs, we propose to enhance existing VA models with elements of another collaborative care management model, Coordinated Anxiety Learning and Management, or CALM. CALM is a flexible, patient preference-driven model for multiple anxiety disorders (panic, generalized anxiety, social anxiety, PTSD) and depression in PC. Evidence for the effectiveness of CALM comes from a large (n = 1004) randomized controlled effectiveness trial, in 17 US primary care clinics with 71% women [15,22]. Women receiving collaborative care showed larger reductions in anxiety, greater improvements in mental health functioning, and larger reductions in days of restricted activity than women receiving usual care, while men showed no differences in these measures. Women receiving the computer-assisted CBT (which used disorder-specific treatment modules) attended more sessions of psychotherapy, completed more modules of therapy, expressed more commitment, and viewed psychotherapy as more helpful than did men [23]. Although Veterans were not the focus of the CALM trial, past trials using cognitive behavioral strategies for anxiety and depression in VA PC clinics have demonstrated the value of the general approach [24]. Importantly, the effectiveness of CALM was strongly related to level of engagement; outcome was predicted by completing exposures and homework and attending more frequently [25]. CALM’s care management components closely mirror those emphasized in VA-approved models described above, including screening and initial assessments using structured instruments; regular monitoring of treatment adherence and side effects using a standardized protocol; patient education and activation; decision support using a protocol to determine patient preference for medication vs a brief computer-assisted CBT-based therapy or both; a stepped care protocol supported by regular brief assessments and a patient tracking system; and assistance with referral to specialty care when appropriate.

*Procedures*

Implementation Strategy Overview. We will use the REP implementation strategy, which involves a phased approach to implementation. Furthermore, complexity theory will enhance the theoretical foundation of REP, and the TECH tool will be used in this project to assess and evaluate implementation challenges. Complexity theory is an appropriate implementation theory for this project because, as described above, both women’s health PC and MH in VA represent “layers of complexity” [26] within the complex adaptive system of the VA. Both of these clinical arenas vary substantially across VAs in terms of their care configurations, resources, space, and interconnectedness. Given this heterogeneity, it is inevitable that challenges will arise during the course of implementation. Our engagement with multiple stakeholders during the course of implementation will provide us with consistent opportunities to “make sense” of and address these challenges, using the techniques provided by the TECH [26].

As described in detail below, at each site, according to the REP framework, we will locally tailor CCWV, provide training and technical assistance, systematically document and address implementation challenges, re-customize model delivery as the need arises, and distill implementation successes that will promote spread of the model.

*Study Design*

We will use a modified stepped wedge design to evaluate the implementation in four VA Women’s PBRN sites. In the context of the modified stepped wedge design, the intervention is “turned on” when a PCP at a site makes her/his first referral to the CCWV care manager (see below).

In Year 1, we will begin the REP pre-conditions phase in two VA Women’s PBRN sites. We will continue through the phases over the course of four years, with a 15-month implementation phase in order to ensure adequate time to evaluate implementation and sufficient numbers of Women Veterans being exposed to CCWV. Two additional PBRN sites will be “stepped in” during Year 2. According to the REP framework and complexity theory, all sites will be encouraged to adapt and tailor set-up and delivery of CALM according to their local resources and care configurations. This means that the “menu options” for adapting delivery that are developed during the pre-conditions phase could differ at each site. Sites 3 and 4 will be aware of the options that were developed and utilized at the first two sites, and may choose to incorporate some of those options as well as options that are available only at their sites.

*REP Implementation Phases*

Pre-conditions (Aim 1): The need for CCWV has been established and the review of effective interventions has been conducted (see above). To ensure that the care model fits local settings and stakeholder priorities, we will visit the sites and meet with the local team to discuss local structures, care models and processes, educational needs, as well as elements of the proposed CCWV and tailoring options that we have conceptualized (Table 3). Using the TECH, the local team will discuss options for CCWV implementation and anticipated barriers to implementation. Also during this phase, interviews and surveys will be conducted with all consenting key stakeholders, with the exception of patients who will be interviewed and surveyed in the implementation phase (see below).

**Table 1. Elements of Collaborative Care for Women Veterans (CCWV)**

| **Care management Functions** | **CCWV Element** | **Local Tailoring Options** |
| --- | --- | --- |
| Initial MH assessment of patients referred by PCP | Care manager conducts baseline MH assessments, medication history, and other relevant information collection | Site designates a local MH care manager (RN or social worker) who conducts some or all care management functions; sites choose from VA-approved assessment packages |
| Women with positive assessments offered treatment choice | Treatment choices for anxiety, depression and PTSD are medications, primary care-based CALM CBT, or both | Site chooses integrated MH providers according to local availability (psychologist; social worker) for training in CALM CBT |
| Women with severity or complexity requiring specialty treatment assisted to access specialty resources | Care manager inquires about preferences for gender-specific services and provider gender; facilitates connection to most congruent locally available specialty services, and makes a warm handoff if possible; care manager follows up with patient to check on any missed appointments or group sessions | Referral can be made to co-located or distant specialty services as available at the site; care manager is familiar with locally available gender-specific groups and providers most experienced in treating women; same gender provider options identified ahead of time |
| Systematic monitoring of symptoms and treatment emergent problems | Care manager uses structured MH assessments for depression, PTSD, anxiety disorders, and alcohol problems as well as symptom and side effects checklists to monitor treatment response; follows up to assure medication and/or CBT session compliance | Sites choose from VA-approved follow-up assessment options; site can choose to involve peer support for telephone check-in with patient and in-person chaperoning and escorting to various services |
| Patient education and activation | If the patient has chosen medication, care manager provides basic patient education and activation relevant to the primary diagnosis; if CBT is chosen the care manager facilitates connection to an integrated provider trained in CALM | Site decides which materials from available care management programs care manager will offer; if in the scope of practice of the locally chosen care manger (e.g., MSW) the care manager may be trained to provide CALM CBT |
| Decision support | Care manager uses a stepped care protocol under supervision of a local psychiatrist to determine when decisions to increase or change treatment options are needed and to collect information to assist the primary care provider in making adjustments | Decision support templates may be locally selected from VA-approved options; sites choose a supervising psychiatrist who may be co-located or based in specialty mental health depending on local circumstances |

Pre-implementation (Aim 1 cont.): At each site, a local CCWV champion will be identified by the Site Lead when s/he signs onto the project. The CCWV package will be further developed and locally tailored, with attention to training and technical assistance needed at each site. Once prepared, the package will be piloted locally to ensure that it works as intended with local systems and processes. Using the TECH, any challenges with pilot implementation will be discussed and addressed by the team. When the team determines that challenges have been sufficiently addressed, orientation meetings will be held with the broader clinic, where the CCWV package will be distributed, discussed, and marketed.

Implementation (Aim 2): The implementation phase will begin at two PBRN sites in the latter half of Year 2 of the EMPOWER QUERI Program and will last a total of 15 months to ensure adequate time for patient exposure to the care model as well as implementation evaluation and reach. CCWV will spread to two additional PBRN sites in Year 3. Care managers will be trained in CALM by CALM experts. Field notes will be taken during the training and minutes will be kept of all conference calls and meetings. Notes will be analyzed using ATLAS.ti and will be interpreted in conjunction with other evaluation data as described below. During regular implementation meetings, TECH will be used to assess and address implementation challenges.

Procedures: Once training is complete and sites are ready for implementation, women Veterans with possible depression, PTSD and/or anxiety disorders will be referred by their PCP (or other referring provider) to the locally identified CCWV care manager, who may also conduct local case finding activities to identify women Veterans who may benefit from care management. The care manager will then complete a clinical evaluation to identify the disorder of primary clinical focus. CCWV is an innovative care model that is proactive, patient-centered and personalized to each woman that has an appointment with the care manager. Specifically, the care manager assists the patient in deciding which type of treatment she wants to pursue (CBT, medication, both or neither). During or shortly after the initial meeting with the care manager, each woman will be asked by the RA if she is willing to participate in our research project, meaning that she will be asked to complete a brief battery of measures at baseline and at six-months post-baseline. Every third woman will also be asked if she is willing to participate in a brief qualitative interview at baseline and six-month follow-up.

For women who choose CBT, the CALM-trained provider will then meet with the patient weekly to review the CALM CBT modules. The program includes five general modules (introduction, psychoeducation, self-monitoring, breathing retraining and relapse prevention), and three other modules that are customized to each anxiety disorder and PTSD (cognitive restructuring, in vivo exposure and interoceptive exposure). Depression modules can also be selected for women with such symptoms. The CALM CBT platform is a computer-assisted delivery system. The provider sits with the patient in front of the computer and directs the individual to review the appropriate material, clarifying and individually tailoring. The program contains educational material as well as skills training and exposure exercises. The entire program is typically completed within eight 60-minute sessions [27]. Consistent with a study of CALM engagement and retention, a woman will be considered to have been “retained” in the care model if she completes six of the eight sessions.

Maintenance and Evolution: The last phase in this framework allows us to take the feedback and make modifications to our implementation process to enhance CCWV adoption, fidelity to the REP framework, and dissemination to future sites. Sharing these details with stakeholders using TECH informs the current implementation process and sustainability for the future. The four REP phases allow us to account for each step of the guided implementation strategy. Finally, the interchange between these phases and the implementation process components provide an in-depth understanding of whether CCWV implementation requires further adaptation and customization prior to broader VA dissemination. During this phase, the research team will collaborate with the local implementation teams to develop points for the CCWV Implementation Playbook.

*Implementation Evaluation*

Mixed Methods Data Collection. Mixed methods will be used to evaluate CCWV implementation (Aim 3). Key stakeholder (KS) semi-structured interviews will be conducted at three time points the sites with WH PCPs, PC-MHI team members, mental health providers, and administrators (~15 KS per site) to assess perceived (pre-conditions) and actual feasibility and acceptability of and satisfaction with CCWV. At the first interview, they will also be asked to complete the organizational and provider measures.

We will collect baseline patient information by asking each woman (n=130) to participate in our project after her appointment with the care manager. Those who agree will complete informed consent and then be asked to complete a survey after the appointment or at another convenient time. Those who complete the baseline survey will receive $10. They will be asked for contact information in order to reach them for a six-month follow-up survey ($10). A subset of patients (approximately 45 per site, or every 3^rd^ consented patient) will be asked to participate in a brief semi-structured interview at baseline ($10) and six months post-baseline ($10). As part of care management, women’s symptoms and functioning will be tracked using the locally available tracking system (e.g., BHL). We will obtain access to that information to augment our analyses.

Measures. Implementation outcomes for this project will be consistent with the implementation outcomes for all projects, i.e., adoption, acceptability, feasibility, and penetration/reach. Additional project-specific measures will also be included. For providers, interview questions will be designed to understand how CCWV impacts the ease with which women’s anxiety, PTSD and/or depression are managed, the impact of the model on addressing more complex MH issues (e.g., stepping a patient up to a higher level of care if warranted), the ways in which women’s psychosocial issues impact ability to remain in the care model, coordination issues with specialty mental health, relative priority and compatibility of CCWV within the general workflow, and competing demands, as well as other barriers and facilitators associated with CCWV implementation.

For patients, project-specific baseline interview questions will focus on women’s history of MH care in VA, their comfort with and degree of preference for MH care in the primary care context, their challenges (if any) with obtaining MH care, and their perception of the importance of being asked to choose what type of MH treatment they want for their anxiety, PTSD and/or depression. Six-month follow-up interviews will focus on women’s experience in the care model, their challenges (if any) with completing the CBT modules (if they selected CBT), their experiences of coordinating their PC and MH care, and their recommendations for caring for women Veterans with mental health concerns.

*Analysis*

Qualitative Analysis*:* In addition to the general EMPOWER QUERI approach to qualitative analysis, for providers, we will compare and contrast data from those who referred to the care manager frequently versus infrequently (or never), and track changes in perceptions about the utility and impact of CCWV on clinical practice from pre-conditions to maintenance. We will also be interested in the experiences of the care managers and other stakeholders themselves with regard to working specifically with women, eliciting their preferences, providing the CBT, and coordinating with MH providers. For patients, we will compare and contrast data from those who selected different CCWV components (CBT, medication, or both). In order to evaluate the patient impact of CCWV, we will assess changes in patient perceptions of their MH symptoms, self-efficacy, and activation.

Quantitative Analysis: We will use the modified stepped wedge design to evaluate the implementation across the providers at four sites. CCWV will be defined as “turning on” when a provider refers a patient to the CCWV care manager. We will model the effect of CCWV implementation on referral rates, while controlling for organizational level, provider level and patient level covariates.

*Impact*

Addressing the treatment needs of women with depression and anxiety is a key strategic priority for our operations partners. These policy partners have specifically identified a need for novel, gender-sensitive primary-care based treatment models for women with these relatively common and understudied mental health problems. They will use our findings to inform policy and practice in MH care for women Veterans. Furthermore, we hope that the sites will find the collaborative care training and implementation useful additions to their arsenal of services for women Veterans in need of MH care in primary care settings. Should implementation be successful, we have many opportunities, through our partners and research team, for scale up and spread of the innovative care model.

**References**

1. Hamilton A, NC M, Oishi S, et al. Mental health needs and diagnoses among women Veterans receiving Veterans Health Administration health care. 2014.

2. Maguen S, Ren L, Bosch JO, Marmar C, Seal KH. Gender differences in mental health diagnoses among Iraq and Afghanistan veterans enrolled in Veterans Affairs health care. Am. J. Public Health. 2010;100:2450–6.

3. Kimerling R, Gima K, Smith MW, Street A, Frayne S. The Veterans Health Administration and Military Sexual Trauma. Am. J. Public Health. 2007;97:2160–6.

4. Valdez C, Kimerling R, Hyun JK, Mark HF, Saweikis M, Pavao J. Veterans Health Administration Mental Health Treatment Settings of Patients Who Report Military Sexual Trauma. J. Trauma Dissociation. 2011;12:232–43.

5. Dichter ME, Cerulli C, Bossarte RM. Intimate Partner Violence Victimization Among Women Veterans and Associated Heart Health Risks. Womens Health Issues. 2011;21:S190–S194.

6. Oishi SM, Rose DE, Washington DL, MacGregor C, Bean-Mayberry B, Yano EM. National Variations in VA Mental Health Care for Women Veterans. Womens Health Issues. 2011;21:S130–S137.

7. Gilbody S. Collaborative Care for Depression: A Cumulative Meta-analysis and Review of Longer-term Outcomes. Arch. Intern. Med. 2006;166:2314.

8. Adli M, Bauer M, Rush AJ. Algorithms and Collaborative-care Systems for Depression: Are They Effective and Why? Biol. Psychiatry. 2006;59:1029–38.

9. Thota AB, Sipe TA, Byard GJ, Zometa CS, Hahn RA, McKnight-Eily LR, et al. Collaborative Care to Improve the Management of Depressive Disorders. Am. J. Prev. Med. 2012;42:525–38.

10. VHA Handbook. 1160.01. Uniform mental health services in VA Medical Centers and Clinics. Washington DC: Department of Veterans Affairs, Veterans Health Administration; 2008.

11. Tew J, Klaus J, Oslin DW. The behavioral health laboratory: Building a stronger foundation for the patient-centered medical home. Fam. Syst. Health. 2010;28:130–45.

12. Pomerantz AS, Shiner B, Watts BV, Detzer MJ, Kutter C, Street B, et al. The White River model of colocated collaborative care: A platform for mental and behavioral health care in the medical home. Fam. Syst. Health. 2010;28:114–29.

13. Chaney EF, Rubenstein LV, Liu C-F, Yano EM, Bolkan C, Lee M, et al. Implementing collaborative care for depression treatment in primary care: A cluster randomized evaluation of a quality improvement practice redesign. Implement. Sci. [Internet]. 2011 [cited 2017 May 12];6. Available from: http://implementationscience.biomedcentral.com/articles/10.1186/1748-5908-6-121

14. Integrating Mental Health Into PACT (IMHIP). Report on Integrating Mental Health Into PACT in the VA. VA Health Services Research & Development, VA Office of Patient Care Services; 2013.

15. Roy-Byrne P, Craske MG, Sullivan G, Rose RD, Edlund MJ, Lang AJ, et al. Delivery of Evidence-Based Treatment for Multiple Anxiety Disorders in Primary Care: A Randomized Controlled Trial. JAMA. 2010;303:1921.

16. VA/DoD Clinical Practice Guideline for the Management of Posttraumatic Stress. Washington, D.C.: Management of Posttraumatic Stress Working Group, Office of Performance and Quality, VA, Quality Management Directororate, US Army MEDCOM; 2010.

17. Kimerling R, Pavao J, Greene L, Karpenko J, Rodriguez A, Saweikis M, et al. Access to Mental Health Care Among Women Veterans: Is VA Meeting Women’s Needs? Med. Care. 2015;53:S97–S104.

18. Wells KB, Sherbourne C, Schoenbaum M, Duan N, Meredith L, Unützer J, et al. Impact of disseminating quality improvement programs for depression in managed primary care: a randomized controlled trial. JAMA. 2000;283:212–20.

19. Unützer J, Katon W, Callahan CM, Williams, Jr JW, Hunkeler E, Harpole L, et al. Collaborative Care Management of Late-Life Depression in the Primary Care Setting: A Randomized Controlled Trial. JAMA. 2002;288:2836.

20. Engel CC, Oxman T, Yamamoto C, Gould D, Barry S, Stewart P, et al. RESPECT-Mil: Feasibility of a systems-level collaborative care approach to depression and post-traumatic stress disorder in military primary care. Mil. Med. 2008;173:935–40.

21. Post EP, Metzger M, Dumas P, Lehmann L. Integrating mental health into primary care within the Veterans Health Administration. Fam. Syst. Health. 2010;28:83–90.

22. Craske MG, Stein MB, Sullivan G, Sherbourne C, Bystritsky A, Rose RD, et al. Disorder-Specific Impact of Coordinated Anxiety Learning and Management Treatment for Anxiety Disorders in Primary Care. Arch. Gen. Psychiatry. 2011;68:378.

23. Grubbs KM, Cheney AM, Fortney JC, Edlund C, Han X, Dubbert P, et al. The Role of Gender in Moderating Treatment Outcome in Collaborative Care for Anxiety. Psychiatr. Serv. 2015;66:265–71.

24. Lang AJ, Norman GJ, Casmar PV. A randomized trial of a brief mental health intervention for primary care patients. J. Consult. Clin. Psychol. 2006;74:1173–9.

25. Glenn D, Golinelli D, Rose RD, Roy-Byrne P, Stein MB, Sullivan G, et al. Who gets the most out of cognitive behavioral therapy for anxiety disorders? The role of treatment dose and patient engagement. J. Consult. Clin. Psychol. 2013;81:639–49.

26. Simpson KM, Porter K, McConnell ES, Colón-Emeric C, Daily KA, Stalzer A, et al. Tool for evaluating research implementation challenges: A sense-making protocol for addressing implementation challenges in complex research settings. Implement. Sci. [Internet]. 2013 [cited 2017 May 11];8. Available from: http://implementationscience.biomedcentral.com/articles/10.1186/1748-5908-8-2

27. Sullivan G, Craske MG, Sherbourne C, Edlund MJ, Rose RD, Golinelli D, et al. Design of the Coordinated Anxiety Learning and Management (CALM) study: innovations in collaborative care for anxiety disorders. Gen. Hosp. Psychiatry. 2007;29:379–87.
